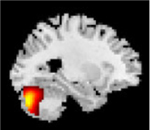# Mode Coresets for Efficient, Interpretable Tensor Decompositions: An Application to Feature Selection in fMRI Analysis

**DOI:** 10.1109/access.2024.3517338

**Published:** 2024-12-13

**Authors:** BEN GABRIELSON, HANLU YANG, TRUNG VU, VINCE CALHOUN, TÜLAY ADALI

**Affiliations:** 1 Department of Computer Science and Electrical Engineering, University of Maryland, Baltimore County, Baltimore, MD 21250, USA; 2 Tri-Institutional Center for Translational Research in Neuroimaging and Data Science (TReNDS), Georgia State University, Atlanta, GA 30302, USA; 3 Georgia Institute of Technology, Emory University, Atlanta, GA 30322, USA

**Keywords:** Tensor decomposition, tucker decomposition, higher order singular value decomposition, coresets, tensor CUR decomposition, subset selection, feature selection, fMRI

## Abstract

Generalizations of matrix decompositions to multidimensional arrays, called *tensor decompositions*, are simple yet powerful methods for analyzing datasets in the form of tensors. These decompositions model a data tensor as a sum of rank-1 tensors, whose *factors* provide uses for a myriad of applications. Given the massive sizes of modern datasets, an important challenge is how well computational complexity scales with the data, balanced with how well decompositions approximate the data. Many efficient methods exploit a small subset of the tensor’s elements, representing most of the tensor’s variation via a basis over the subset. These methods’ efficiencies are often due to their randomized natures; however, deterministic methods can provide better approximations, and can perform *feature selection*, highlighting a meaningful subset that well-represents the entire tensor. In this paper, we introduce an efficient subset-based form of the Tucker decomposition, by selecting *coresets* from the tensor modes such that the resulting core tensor can well-approximate the full tensor. Furthermore, our method enables a novel feature selection scheme unlike other methods for tensor data. We introduce methods for random and deterministic coresets, minimizing error via a measure of discrepancy between the coreset and full tensor. We perform the decompositions on simulated data, and perform on real-world fMRI data to demonstrate our method’s feature selection ability. We demonstrate that compared with other similar decomposition methods, our methods can typically better approximate the tensor with comparably low computational complexities.

## I. INTRODUCTION

Datasets in the modern era often take the form of large multidimensional arrays called *tensors*. A tensor can be understood as a collection of values (e.g. measurements) that are each associated with a corresponding list of N array indices, where N denotes the order of the tensor. Whereas a *vector* is a first order tensor and a *matrix* is a second order tensor, the analysis of third or higher order tensors is the focus of those methods formally called tensor decompositions. Tensor decompositions generalize matrix decompositions to higher order tensors, approximating a tensor dataset as a tensor product of several factor matrices that have various use cases. These generalizations notably endow tensor decompositions with the ability to model *multilinear* relationships within the data, concisely modeling the relationships across different modes of the tensor. Furthermore, tensor decompositions provide a low-rank model of the tensor that typically is orders of magnitude smaller in memory than the original tensor. A tensor decomposition’s factors are typically useful for describing the latent characteristics of the tensor, and are often used for providing a generative model of the data. All in all, tensor decompositions provide tools for a wide range of uses, such as dimension reduction [1], [2], [3], [4], [5], feature extraction [6], [7], [8], [9], denoising [10], [11], [12], [13], [14], [15], missing data completion [15], [16], [17], [18], [19], [20], dictionary learning [21], [22], [23], [24], [25], signal processing [26], [27], [28], [29], [30], [31], [32], and various others. Applications of tensor decompositions are widespread and include chemometrics [1], [33], [34], psychometrics [35], [36], econometrics [37], [38], analysis of medical imaging modalities [7], [39], [40], [41], [42], [43], [44], [45], [46], [47], [48], radar and communication applications [30], [49], [50], applications to machine learning [26], [51], [51], [52], [53], [54], [55], [56], and many others.

Perhaps one of the simplest tensor decompositions is what is often called the canonical polyadic decomposition (CPD) [57], [58], which approximates a tensor as the sum of R rank-1 tensors where R is a user-defined positive integer. CPD can be understood as a higher-order generalization of matrix low-rank decompositions, which decompose a matrix into a sum of rank-1 matrices that best approximates the original matrix. However, whereas matrix rank decompositions are typically not unique unless additional constraints are imposed, the CPD is often unique under much milder conditions. This results in unique factors that reveal the latent structure of the data under fewer required assumptions [1], [59], [60], [61].

Another useful form of tensor decomposition is the Tucker Decomposition [62], [63]. The Tucker decomposition is a general form of tensor decomposition that represents an Nth order tensor as the tensor product of N factor matrices with an Nth order ‘‘core’’ tensor: a small tensor that can be considered a compressed version of the original tensor. A notable specific type of Tucker decomposition is the higher-order singular value decomposition (HOSVD) [63], [64], the direct generalization of the matrix singular value decomposition (SVD) to tensors. HOSVD is analytically represented by its factor matrices being the singular vectors of each ‘‘unfolding’’ (matricization) of the original tensor, in which case the core tensor can be interpreted as a tensorial form of principal components. While the CPD and Tucker are perhaps the most popularly used tensor decompositions, since their introduction a wide variety of other decompositions have been introduced and used successfully. These include the Tensor Train decomposition [65], [66], hierarchical Tucker decompositions [67], [68], tensor block-term decompositions [69], [70], coupled matrix-tensor factorizations [44], [71], [72], and online tensor decompositions [73], [74], [75], [76].

Most tensor decompositions perform their optimization routines by breaking the problem of estimating all N factor matrices into N simpler subproblems. This typically involves solving for each factor matrix one at a time, by unfolding the tensor with respect to each of the N modes and subsequently solving for (or updating) a corresponding mode’s factor matrix. While these routines simplify optimization by allowing decompositions to be solved with matrix-based methods, tensor decompositions nevertheless rely on multiplying high-dimensional matrix representations of the tensor data, which can become computationally expensive with exceedingly massive tensors. These challenges have greatly motivated computationally efficient methods for tensor decompositions, especially those that retain simple models with excellent approximation and explainability.

Many efficient tensor decompositions are direct generalizations of matrix decompositions. With matrices, a particularly useful strategy has been to approximate a matrix via projecting onto the span of only a small subset of columns. These are referred to as column subset selection (CSS) methods, of which include the matrix CUR decomposition [77], [78] which approximates a given matrix by both a subset of columns C and a subset of rows R. Subset selection methods are distinguished by those that select a subset randomly, with a focus on faster decompositions, or those that select a subset deterministically, with a focus on better approximation and for performing *feature selection*: identifying particularly representative elements of the data that well-describe the rest of the data. Extensions of these matrix decompositions to tensors exist as types of Tucker decompositions that are called tensor CUR decompositions [79], [80], [81], [82], [83], [84], which use subsets of elements from multiple modes of a tensor to provide a multilinear basis for the entire tensor. Due to their simple procedures, tensor CUR decompositions are among the fastest tensor decompositions, and can also provide good approximations of tensors with reasonably large subset sizes, yet may suffer with smaller subset sizes. These methods exclusively select subsets randomly, rather than deterministically. Extensions of deterministic subset-based methods may also be desirable for tensors, especially in the interest of determining well-representative subsets of the data.

Tensorial feature selection has been accomplished in [85], [86], and [87] but only in the context of supervised learning for classification, where tensors are accompanied by labels and feature selection is a function of the labels. An unsupervised feature selection method for third order tensors was proposed in [88], which takes subsets from a single mode of the tensor. However, features in these subsets differ depending on what elements they correspond to in another ‘‘view’’ mode, and thus may be harder to interpret. Furthermore, these subsets are acquired after performing a CPD, whereas the methods we consider in this paper actually use the subsets to perform efficient tensor decompositions. To our knowledge there have been no other extensions of deterministic subset-based methods to tensor data in the general unsupervised setting, and none for multiple modes of the tensor.

In this paper, we introduce an efficient weighted subset-based type of Tucker decomposition, similar in form to the tensor CUR decompositions and the sequentially truncated higher-order singular value decomposition (ST-HOSVD) [89]. Notably, the deterministic variation of our method provides a novel unsupervised feature selection algorithm for tensor data, selecting subsets from one or more modes that are reasonably best able to summarize the structure of the tensor. Sequentially across a tensor’s N modes, we select from each mode a *coreset*, i.e. a weighted subset of elements [90], [91], [92], [93], [94], that reasonably minimizes a measure of discrepancy between the coreset and the entire mode, which in turn minimizes the mean squared error cost between the tensor and its approximation. We connect the discrepancy to the cost function of HOSVD, showing that use of weighed subsets provides a better minimization to the cost than unweighted subsets used in tensor CUR decompositions. We consider two methods: one based on random coreset selection, sampling according to a weighted probability distribution, and one based on deterministic coreset selection, utilizing an efficient weighted kernel herding (WKH) [95] procedure. For a given coreset, we select the corresponding coreset weights via an efficient nonnegative least squares (NNLS) minimizing the discrepancy between the coreset and the entire mode. We analyze performance of our two methods on large datasets, testing with both simulated data and real functional magnetic resonance imaging (fMRI) data via functional connectivity matrices (FNCs) arranged as a large tensor. Comparing with similar Tucker-type methods, such as variations of the Tensor CUR decomposition and randomized HOSVD methods, we demonstrate that our methods are highly efficient, provide good approximation performance, and can be converted to a HOSVD decomposition with strong estimation quality.

The paper is organized as follows. Section II introduces preliminary concepts regarding matrices and tensors, including several basic methods for matrix and tensor decompositions. Section III explains efficient generalizations of subset-based methods for matrix decompositions to tensor decompositions, such as the tensor CUR decompositions. Section IV introduces our proposed sequential coreset-based tensor decomposition methods, which we refer to as tensor coreset decompositions (TCD). Section V provides results of our methods, compared with various other methods, on both simulated tensor datasets and a real fMRI FNC tensor dataset. Section VI concludes the paper and overviews the contributions.

## II. PRELIMINARIES

### A. NOTATION

Throughout the paper, we use notation that is summarized in Table 1, and is consistent with notation of other works that discuss tensor decompositions (e.g., [1]).

We denote scalars by lowercase unbolded letters (e.g., x), vectors by lowercase bolded letters (e.g., x), matrices by uppercase bolded letters (e.g. X), and higher order tensors (order three or higher) by calligraphic bolded letters (e.g. 𝓧).

The *order* of a tensor, N, also referred to as the number of *modes*, can be loosely thought of as the number of dimensions in the tensor, but more precisely it is the number of indices needed to index an entry in the tensor. For instance, a third order tensor 𝓧 has a corresponding $\left(i_{1},i_{2},i_{3}\right)$ element denoted by ${\left(𝓧\right)}_{\left(i_{1}i_{2}i_{3}\right)}$. Each index corresponds to a different mode of the tensor, and is bounded by the *dimensionality* of that mode. For example, given a third-order tensor $𝓧\in {\mathbb{R}}^{D_{1}\times D_{2}\times D_{3}}$, the dimensionality of the first mode is $D_{1}$. In general, when dealing with Nth-order tensors, we refer to the dimensionality of the nth node by $D_{n}$, and a particular index from that mode by $i_{n}$, for $i_{n}=1,\ldots ,D_{n}$, and n=1,…,N.

As our paper utilizes subsets of the tensor, we define a *subtensor* as a subset of elements in the tensor corresponding to some set of indices. We define index sets over a given nth mode of a tensor by unbolded calligraphic letters ${𝓘}_{n}$, and use a colon to otherwise indicate all elements of a mode. For example, ${\left(𝓧\right)}_{\left(i,:,:\right)}$ denotes the ith element of the first mode, and ${\left(𝓧\right)}_{\left({𝓘}_{1},:,:\right)}$ denotes a subset of elements in the first mode corresponding to the index set ${𝓘}_{1}$.

An important operation in tensor decompositions is the matricization of a tensor, also called the unfolding. We denote the nth mode unfolding of a tensor $𝓧\in {\mathbb{R}}^{D_{1}\times D_{2}\times \ldots \times D_{N}}$ by the matrix ${\text{X}}_{\left(n\right)}\in {\mathbb{R}}^{D_{n}\times {\tilde{D}}_{n}}$, where ${\tilde{D}}_{n}=\prod_{\begin{array}{l} m=1\\ m\neq n \end{array}}^{N}D_{m}$ is the product of all other mode’s dimensionalities. The ith row of the nth mode unfolding is the vectorization of the ith element in the nth mode, e.g. ${\left({\text{X}}_{\left(1\right)}\right)}_{\left(3,:\right)}$ denotes the third row in the first mode unfolding of X and is equal to vec ${\left({𝓧}_{\left(3,:,:,\ldots ,:\right)}\right)}^{⊤}$, the third element of the first mode.

The *rank* of a tensor 𝓧 is defined as the smallest number of rank-1 tensors that exactly sum to 𝓧. Unlike with matrices, determining the tensor rank is difficult for most real-world tensors. A more well-defined notion of a tensor’s rank structure are the *n-ranks*, the ranks of each unfolding ${\text{X}}_{\left(n\right)}$.

If the nth mode unfolding of a tensor ${\text{X}}_{\left(n\right)}\in {\mathbb{R}}^{D_{n}\times {\tilde{D}}_{n}}$ is left multiplied by a matrix $\text{U}\in {\mathbb{R}}^{J_{n}\times D_{n}}$, the resulting product $\text{G}\;\text{=}\;\text{U}\;{\text{X}}_{\left(n\right)}\in {\mathbb{R}}^{J_{n}\times {\tilde{D}}_{n}}$ is equivalently represented in the tensor domain by the nth mode tensor product $𝓖\;\text{=}\;𝓧\times_{n}\text{U}\in {\mathbb{R}}^{D_{1}\times \ldots \times D_{n-1}\times J_{n}\times D_{n+1}\times \ldots \times D_{N}}$.

The norm of a tensor $𝓧\in {\mathbb{R}}^{D_{1}\times \ldots \times D_{N}}$ is defined by:

$${\left\|𝓧\right\|}_{\text{F}}={\left(\sum_{i_{1}=1}^{D_{1}}\cdots \sum_{i_{N}=1}^{D_{N}}{\left(𝓧\right)}_{\left(i_{1},\ldots ,i_{N}\right)}^{2}\right)}^{\frac{1}{2}}$$

In the next subsections, we first discuss Tucker and HOSVD decompositions for tensors, and note their complexities. We then discuss column subset selection (CSS) methods for reducing complexities of matrix decompositions, and then discuss generalizations of these methods to tensors.


$$\bar{\underline{\begin{array}{l} \underline{\text{Algorithm 1 HOSVD}\;\;\;\;\;\;\;\;\;\;\;\;\;\;\;\;\;\;\;\;\;\;\;\;\;\;\;\;\;\;}\\ \text{Input:}\;𝓧\in {\mathbb{R}}^{D_{1}\times \ldots \times D_{N}}\;\text{(}N\text{-mode}\;\text{tensor),}\\ \;\;\;[{\hat{R}}_{1},\;\ldots ,\;{\hat{R}}_{N}]\;\text{(number}\;\text{of}\;\text{factors}\;\text{per}\;\text{mode)}\\ \text{Output:}\;𝓧\approx 𝓖\times_{1}{\text{A}}_{1}\times \ldots \times_{M}{\text{A}}_{N},\;\text{where}\\ \;\;\;\;𝓖\in {\mathbb{R}}^{{\hat{R}}_{1}\times \ldots \times {\hat{R}}_{N}}\text{(core}\;\text{tensor),}\\ \;\;\;\;\{{\text{A}}_{1},\;\ldots ,\;{\text{A}}_{N}\}\;\text{(factor}\;\text{matrices)}\\ \;\text{for}\;\text{each}\;\text{mode}\;n=1\;:\;N\\ \;\;\text{unfold}\;\text{(matricize)}\;\text{tensor}\;\text{w.r.t.}\;n\text{th}\;\text{mode}\\ \;\;\;𝓧\to {\text{X}}_{\left(n\right)}\in {\mathbb{R}}^{D_{n}\times {\tilde{D}}_{n}},\;\text{with}\;{\tilde{D}}_{n}=\prod_{\begin{array}{l} m=1\\ m\neq n \end{array}}^{N}D_{m}\\ \;\;\text{compute}\;{\text{A}}_{n}\in {\mathbb{R}}^{D_{n}\times {\hat{R}}_{n}},\\ \;\;\;\text{the}\;{\hat{R}}_{n}\;\text{left}\;\text{singular}\;\text{vectors}\;\text{of}\;{\text{X}}_{\left(n\right)}\\ \;\text{end}\;\text{for}\\ \;𝓖\;\text{=}\;𝓧\times_{1}{\text{A}}_{1}^{⊤}\times_{2}\ldots \times_{N}{\text{A}}_{N}^{⊤} \end{array}}}$$

### B. TUCKER AND HOSVD DECOMPOSITIONS

The Tucker decomposition [62], [63] is a general type of tensor decomposition that approximates an Nth order tensor $𝓧\in {\mathbb{R}}^{D_{1}\times \ldots \times D_{N}}$ by the tensor product of N factor matrices ${\text{A}}_{n}(n=1,\ldots N)$, with a smaller core tensor 𝓖. The general cost function for Tucker decompositions takes the form:

(1) $$𝓙(𝓖,{\text{A}}_{1},\;\ldots ,\;{\text{A}}_{N})={\left\|𝓧-𝓖\times_{1}{\text{A}}_{1}\times_{2}\ldots \times_{N}{\text{A}}_{N}\right\|}_{\text{F}}^{2}$$

where ${\text{A}}_{n}\in {\mathbb{R}}^{D_{n}\times {\hat{R}}_{n}}$ is the nth mode’s factor matrix, $𝓖\in {\mathbb{R}}^{{\hat{R}}_{1}\times \ldots \times {\hat{R}}_{N}}$ is the core tensor, and ${\hat{R}}_{n}$ are the number of factors chosen for the nth mode, which are often closely related to the tensor’s *n*-ranks $R_{n}$, for n=1,…,N.

The Tucker decomposition is not unique without any further constraints. There are a variety of ways to achieve a unique Tucker decomposition over a tensor, including several subset-based approaches such as the tensor CUR decomposition and the method that we later propose in this paper.


$$\bar{\underline{\begin{array}{l} \underline{\text{Algorithm}\;\text{2}\;\text{ST-HOSVD}\;\;\;\;\;\;\;\;\;\;\;\;\;\;\;\;\;\;\;\;\;\;\;\;\;\;}\\ \text{Input:}\;𝓧\in {\mathbb{R}}^{D_{1}\times \ldots \times D_{N}}\;\text{(}N\text{-mode}\;\text{tensor),}\\ \;\;\;[{\hat{R}}_{1},\;\ldots ,\;{\hat{R}}_{N}]\;\text{(number}\;\text{of}\;\text{factors}\;\text{per}\;\text{mode)}\\ \text{Output:}\;𝓧\approx 𝓖\times_{1}{\text{A}}_{1}\times \ldots \times_{M}{\text{A}}_{N},\;\text{where}\\ \;\;\;\;𝓖\in {\mathbb{R}}^{{\hat{R}}_{1}\times \ldots \times {\hat{R}}_{N}}\text{(core}\;\text{tensor),}\\ \;\;\;\;\{{\text{A}}_{1},\;\ldots ,\;{\text{A}}_{N}\}\;\text{(factor}\;\text{matrices)}\\ \;\text{for}\;\text{each}\;\text{mode}\;n=1\;:\;N\\ \;\;\text{unfold}\;\text{(matricize)}\;\text{tensor}\;\text{w.r.t.}\;n\text{th}\;\text{mode}\\ \;\;\;𝓧\to {\text{X}}_{\left(n\right)}\in {\mathbb{R}}^{D_{n}\times {\left({\tilde{D}}_{n}\right)}^{\left(n\right)}},\\ \;\;\;\text{with}\;{\left({\tilde{D}}_{n}\right)}^{\left(n\right)}=(\prod_{m=1}^{n-1}{\hat{R}}_{m})\;(\prod_{m=n+1}^{n-1}D_{m})\\ \;\;\text{compute}\;{\text{A}}_{n}\in {\mathbb{R}}^{D_{n}\times {\hat{R}}_{n}},\\ \;\;\;\text{the}\;{\hat{R}}_{n}\;\text{left}\;\text{singular}\;\text{vectors}\;\text{of}\;{\text{X}}_{\left(n\right)}\\ \;\;\text{truncate}\;\text{the}\;\text{unfolded}\;\text{tensor}\\ \;\;\;{\text{X}}_{\left(n\right)}\to {\text{A}}_{n}^{⊤}{\text{X}}_{\left(n\right)}\in {\mathbb{R}}^{{\hat{R}}_{n}\times {\left({\tilde{D}}_{n}\right)}^{\left(n\right)}}\\ \;\;\text{un-matricize the tensor}\\ \;\;\;{\text{X}}_{\left(n\right)}\to 𝓧\in {\mathbb{R}}^{{\hat{R}}_{1}\times \ldots \times {\hat{R}}_{n}\times D_{n+1}\times \ldots \times D_{n}}\\ \;\text{end}\;\text{for}\\ \;𝓖\;\to \;𝓧 \end{array}}}$$

A useful Tucker decomposition is the HOSVD [63], [64], a natural generalization of SVD to tensors. HOSVD’s factor matrices of a tensor 𝓧 are analytically given as the left singular vectors of each unfolding of 𝓧, and the core tensor is obtained from a tensor product of these factor matrices with 𝓧. The HOSVD procedure is described in Algorithm 1.

Several variations of HOSVD have been introduced since its inception to improve its efficiency, with one of the most used variations being the sequentially truncated HOSVD (ST-HOSVD) [89]. Across each nth mode of the tensor, ST-HOSVD first estimates a mode’s factor matrix from the left singular vectors of the nth mode unfolding ${\text{X}}_{\left(n\right)}$ (just as done with HOSVD), and then replaces 𝓧 with the core tensor formed by the tensor product of this factor matrix with 𝓧. Over calculation of the N mode factor matrices, the current tensor progressively reduces in size until it becomes the final core tensor and all N factor matrices are obtained. The ST-HOSVD procedure is described in Algorithm 2.

If we denote the SVD of each ${\text{X}}_{\left(n\right)}$ in the for loop of Algorithm 2 by ${\text{X}}_{\left(n\right)}={\text{U}}_{{\text{X}}_{\left(n\right)}}\sum_{{\text{X}}_{\left(n\right)}}{\text{V}}_{{\text{X}}_{\left(n\right)}}^{⊤}$, such that ${\text{A}}_{n}={\text{U}}_{{\text{X}}_{\left(n\right)}}$, it follows that ST-HOSVD’s truncation strategy sequentially replaces ${\text{X}}_{\left(n\right)}$ with its top ${\hat{R}}_{n}$ right principal components (PCs) $\sum_{{\text{X}}_{\left(n\right)}}{\text{V}}_{{\text{X}}_{\left(n\right)}}^{⊤}$, thus best preserving the approximation of the original tensor while reducing the dimensionality of operations across all remaining modes.

We now compare the computational complexities of HOSVD and ST-HOSVD. For simplicity, we assume that the order of modes truncated with ST-HOSVD is n=1,…,N. HOSVD’s computational complexity is $𝓞(\sum_{n-1}^{N}\min(D_{n}^{2}{\tilde{D}}_{n},{\tilde{D}}_{n}^{2}D_{n}))$, dominated by the N SVD-unfoldings for large tensors. ST-HOSVD considerably reduces this complexity to $𝓞(\sum_{n-1}^{N}\min(D_{n}^{2}{\tilde{D}}_{n}^{\left(n\right)},{\left({\tilde{D}}_{n}^{\left(n\right)}\right)}^{2}D_{n}))$, where ${\tilde{D}}_{n}^{\left(n\right)}=(\prod_{m=1}^{n-1}{\hat{R}}_{m})\;(\prod_{m=n+1}^{N}D_{m})$, here ${\hat{R}}_{m}$ is the number of factors in the mth mode. However with ST-HOSVD, the first few modes’ SVDs are similar in complexity to those calculated with HOSVD. This leads ST-HOSVD to still be computationally expensive when dealing with large tensors, motivating more scalable decomposition methods.

In the next section, we overview subset-based methods for reducing complexity of matrix decompositions, from which we then overview their various generalizations to tensors.

### C. MATRIX DECOMPOSITIONS BY COLUMN SUBSET SELECTION (CSS)

This subsection gives a general overview of column subset selection methods for matrices. For a more detailed discussion of the topic, we refer the reader to [96], [97], [98].

CSS methods approximate a matrix $\text{X}\in {\mathbb{R}}^{M\times N}$ by selecting a subset of columns of the matrix, selecting either randomly or deterministically, and then approximating X by projecting onto the span of the subset. If we denote ${\text{X}}_{{𝓘}_{s}}\;≜\;{\left(\text{X}\right)}_{\left(:,{𝓘}_{s}\right)}\in {\mathbb{R}}^{M\times N_{s}}$ as the matrix formed by a $N_{s}$ subset of columns, corresponding to some index set ${𝓘}_{s}$, the approximation error for some choice of ${\text{X}}_{{𝓘}_{s}}$ is given by:

(2) $$𝓙({𝓘}_{s})={\left\|\text{X}-{\text{X}}_{{𝓘}_{s}}{\left({\text{X}}_{{𝓘}_{s}}^{⊤}{\text{X}}_{{𝓘}_{s}}\right)}^{-1}{\text{X}}_{{𝓘}_{s}}^{⊤}\text{X}\right\|}_{\text{F}}^{2}\;\;=\;{\left\|\text{X}-{\text{X}}_{{𝓘}_{s}}{{\text{X}}_{{𝓘}_{s}}}^{†}\text{X}\right\|}_{\text{F}}^{2}\;\;=\;{\left\|\text{X}-{\text{P}}_{{\text{X}}_{{𝓘}_{s}}}\text{X}\right\|}_{\text{F}}^{2}$$

where ${{\text{X}}_{{𝓘}_{s}}}^{†}={\left({\text{X}}_{{𝓘}_{s}}^{⊤}{\text{X}}_{{𝓘}_{s}}\right)}^{-1}{\text{X}}_{{𝓘}_{s}}^{⊤}$ is the pseudoinverse of ${\text{X}}_{{𝓘}_{s}}$ (such that ${{\text{X}}_{{𝓘}_{s}}}^{†}{\text{X}}_{{𝓘}_{s}}=\text{I}\in {\mathbb{R}}^{N_{s}\times N_{s}}$, and ${\text{P}}_{{\text{X}}_{{𝓘}_{s}}}={\text{X}}_{{𝓘}_{s}}{{\text{X}}_{{𝓘}_{s}}}^{†}\in {\mathbb{R}}^{M\times M}$ is the projection matrix corresponding to the column space of ${\text{X}}_{{𝓘}_{s}}$. This is equivalently given by:

(3) $$𝓙({𝓘}_{s})={\left\|\text{X}-{\text{X}}_{{𝓘}_{s}}{\text{M}}_{{𝓘}_{s}}\right\|}_{\text{F}}^{2}$$

where ${\text{M}}_{{𝓘}_{s}}={\left({\text{X}}_{{𝓘}_{s}}^{⊤}{\text{X}}_{{𝓘}_{s}}\right)}^{-1}{\text{X}}_{{𝓘}_{s}}^{⊤}\;\text{X}\in {\mathbb{R}}^{N_{s}\times N}$ is a matrix mapping columns of X onto the span of ${\text{X}}_{{𝓘}_{s}}$, which in later sections we refer to as a ‘‘mapping’’ matrix.

#### 1) RANDOMIZED CSS

Randomized CSS methods operate by assigning a weighted probability distribution to the columns and then sampling according to this distribution. Uniform sampling of the columns (giving equal sampling probability to each column) generally produces bad approximations of a matrix, especially if the columns are heterogeneous. Instead, sampling distributions are often based on probabilities weighted by the squared norm of columns, i.e. ‘‘norm sampling’’ [77], [99], or approximated statistical leverage scores [100]. In our paper, we focus on norm sampling, which is the most computationally efficient of the sampling-based methods, and we note that norm sampling is also conventional in tensor-based methods [79], [82], [101]. It has been proven in [99] that norm sampling provides the following error guarantees: given a matrix $\text{X}\in {\mathbb{R}}^{M\times N}$ and values for ϵ, δ, and a defined upper limit to the rank k of ${\text{P}}_{{\text{X}}_{{𝓘}_{s}}}={\text{X}}_{{𝓘}_{s}}{{\text{X}}_{{𝓘}_{s}}}^{†}$, then a norm sampled selection for ${\text{X}}_{{𝓘}_{s}}$ satisfies the following error probability

$$\mathrm{Pr}\{{\left\|\text{X}-{\text{P}}_{{\text{X}}_{{𝓘}_{s}}}\text{X}\right\|}_{\text{F}}^{2}\leq {\left\|\text{X}-{\text{X}}_{k}\right\|}_{\text{F}}^{2}+\epsilon {\left\|\text{X}\right\|}_{\text{F}}^{2}\}\;\geq \;1-\delta$$

where ${\text{X}}_{k}$ is the best rank-*k* approximation to X, and 0≤δ≤1 is the probability of failure.

Furthermore, it has been proven in [77] that given a norm sampled subset of columns ${\text{X}}_{{𝓘}_{s}}$, after rescaling the columns of ${\text{X}}_{{𝓘}_{s}}$ to be the same norm

(4) $${\left({\text{X}}_{{𝓘}_{s}}\right)}_{\left(:,i\right)}\to \frac{1}{\sqrt{N_{s}}}\frac{{\left\|\text{X}\right\|}_{\text{F}}}{{\left\|{\left({\text{X}}_{{𝓘}_{s}}\right)}_{\left(:,i\right)}\right\|}_{\text{F}}}{\left({\text{X}}_{{𝓘}_{s}}\right)}_{\left(:,i\right)}$$

that the following error probability is satisfied ∀ϵ≥0:

$$\mathrm{Pr}\{{\left\|\text{X}{\text{X}}^{⊤}-{\text{X}}_{{𝓘}_{s}}{\text{X}}_{{𝓘}_{s}}^{⊤}\right\|}_{\text{F}}^{2}\leq \frac{\eta }{{\left(N_{s}\right)}^{\frac{1}{2}}}{\left\|\text{X}\right\|}_{\text{F}}^{2}\}\;\geq \;1-\delta$$

where $\eta =1+{\left(8\log(\delta^{-1})\right)}^{\frac{1}{2}}$.

If we denote the SVD of X by $\text{X}={\text{U}}_{\text{X}}\sum_{\text{X}}{\text{V}}_{\text{X}}^{⊤}$, this particular result suggests that with a high enough sample size $N_{s}$, a norm sampled ${\text{X}}_{{𝓘}_{s}}$ can adequately approximate the left PCs ${\text{U}}_{\text{X}}\sum_{\text{X}}$ of X with a high probability, by re-scaling the columns according to (4). We later will refer to this result when introducing our coreset-based method.

#### 2) DETERMINISTIC CSS

Deterministic CSS methods are combinatorial methods for selecting a ‘‘best’’ representative subset of columns, where ‘‘best’’ is relative to the method used. The problem of finding a subset that exactly minimizes the approximation cost over all possible subsets has been acknowledged as being UG-hard (where ‘‘UG’’ refers to the unique games conjecture) [102], in which case deterministic algorithms mainly focus on obtaining a reasonably ‘‘best’’ subset in a reasonable amount of time. These methods can also effectively serve as *feature selection* methods, and thus there is a large overlap between methods that can be used for feature selection and those used for deterministic CSS. However, the design of CSS methods typically puts a greater emphasis on the scalability of methods, especially with the high-dimensional combinatorial problems posed by large matrices or tensors.

Perhaps the most popular method for deterministic CSS is to use the greedy algorithm, which consecutively searches for a new column to add onto a subset such that the resulting new subset best approximates the full matrix. The greedy CSS algorithm was first studied in [103], and has been demonstrated to be both scalable to large numbers of columns and provide high-quality representative subsets [97], [104], [105], [106], [107], [108].

As one may expect, deterministic CSS methods provide better approximation than randomized methods and incur better error guarantees. The tightest bounds for deterministic CSS depend on the singular values of X; intuitively, those matrices whose singular values have higher rate of decay are simpler matrices which require much fewer columns to well-approximate the matrix. In [98], the following bound was proven on greedy CSS:

$${\left\|\text{X}-{\text{P}}_{{\text{X}}_{{𝓘}_{s}}}\text{X}\right\|}_{\text{F}}^{2}\geq (1-\epsilon ){\left\|\text{X}-{\text{X}}_{k}\right\|}_{\text{F}}^{2}$$

where ${\text{X}}_{k}$ is the best rank-*k* approximation to X, $r\;\geq \;16k{\left(\epsilon \sigma_{\min}({\text{X}}_{k})\right)}^{-1}$ is the number of steps taken by the greedy algorithm, and $\sigma_{\min}({\text{X}}_{k})$ is the smallest singular value of ${\text{X}}_{k}$. Similar results have been proven in Theorem 3 of [109]. A shared result amongst these works is that by only taking slightly more than k columns with greedy CS, the approximated matrix is less than a 1−ϵ factor from the optimal choice of k columns.

## III. SUBSET METHODS GENERALIZED TO TENSORS (METHODS TO APPROXIMATE THE HOSVD)

As tensor decompositions frequently invoke matrix operations with the tensor unfolding, matrix approximation techniques have found great use for accelerating tensor decompositions [79], [80], [81], [82], [83], [101]. As our proposed method is most analogous to the HOSVD, we focus only on those subset-based methods for performing a Tucker decomposition in the form of an approximated HOSVD.

These methods generally estimate a form of Tucker decomposition that is not a HOSVD decomposition, but can be used to approximate one. In order to provide an approximate HOSVD decomposition, we may convert any method’s corresponding Tucker decomposition to a HOSVD decomposition via the procedure [82] outlined in Algorithm 3.


$$\bar{\underline{\begin{array}{l} \underline{\text{Algorithm}\;\text{3}\;\text{Convert}\;\text{Tucker}\;\text{decomposition}\;\text{to}\;\text{HOSVD}}\\ \text{Input:}\;\tilde{𝓖}\in {\mathbb{R}}^{{\hat{R}}_{1}\times \ldots \times {\hat{R}}_{N}}\;\text{(core}\;\text{tensor)}\\ \;\;\;\{{\tilde{A}}_{1},\;\ldots ,\;{\tilde{A}}_{N}\}\;\text{(factor}\;\text{matrices)}\\ \text{Output:}\;𝓖\in {\mathbb{R}}^{{\hat{R}}_{1}\times \ldots \times {\hat{R}}_{N}}\;\text{(HOSVD}\;\text{core}\;\text{tensor),}\\ \;\;\;\;\{{\text{A}}_{1},\;\ldots ,\;{\text{A}}_{N}\}\;\text{(HOSVD}\;\text{factor}\;\text{matrices)}\\ \;\text{for}\;\text{each}\;\text{mode}\;n=1\;:\;N\\ \;\;\text{factorize}\;{\tilde{A}}_{n}\;\text{using}\;\text{the}\;\text{QR}\;\text{decomposition:}\\ \;\;\;[{\text{Q}}_{n},{\text{R}}_{n}]=\text{qr}({\tilde{A}}_{n})\\ \;\;\text{replace}\;\tilde{𝓖}\to \tilde{𝓖}\times_{n}{\text{R}}_{n}\\ \;\text{end}\;\text{for}\\ \;\text{perform}\;\text{HOSVD}\;\text{on}\;\text{the}\;\text{new}\;\text{core}\;\text{tensor:}\\ \;\;\;[𝓖,{\text{A}}_{1},\;\ldots ,\;{\text{A}}_{N}]=\text{HOSVD(}\tilde{𝓖}\text{)}\\ \;\text{for}\;\text{each}\;\text{mode}\;n=1\;:\;N\\ \;\;\text{replace}={\text{A}}_{n}\to {\text{Q}}_{n},{\text{R}}_{n}\\ \;\text{end}\;\text{for} \end{array}}}$$

There are various different strategies to provide a Tucker decomposition over a tensor 𝓧 via exploiting the previously discussed matrix approximation techniques over the tensor unfoldings ${\text{X}}_{\left(n\right)}$. These strategies can generally be separated into two distinct camps with differing decompositions:

- *column-based subsets*: approximate ${\text{X}}_{\left(n\right)}$ by a subset of its columns, e.g. randomized sampling tucker CUR [80]
- *row-based subsets*: approximate ${\text{X}}_{\left(n\right)}$ by a subset of its rows, e.g. Chidori CUR [79], [82], Fiber CUR [81], [82], and randomized-block HOSVD (RB-HOSVD) [101]

We briefly overview and contrast these two strategies in the following subsections.

### A. COLUMN-BASED SUBSET METHODS FOR TENSOR UNFOLDINGS

Column-based subset methods approximate a tensor unfolding ${\text{X}}_{\left(n\right)}$ using a subset of its columns. These columns are referred to as “fibers” in the tensor literature, and represent a fixed index in all modes of the tensor except for the nth mode. As an example, ${\left({\text{X}}_{\left(1\right)}\right)}_{\left(:,z\right)}$ is a fiber of the first mode which represents ${\left(𝓧\right)}_{\left(:,i_{2},i_{3},\ldots ,i_{N}\right)}$ for some indices of the N−1 other modes $i_{2},i_{3},\ldots ,i_{N}$ that correspond to some fiber index z.

For the nth mode unfolding ${\text{X}}_{\left(n\right)}$ of a tensor 𝓧, if we denote ${𝓘}_{n}$ as an index set for some subset of ${\hat{R}}_{n}$ columns, and denote ${\left({\text{X}}_{\left(n\right)}\right)}_{\left({𝓘}_{n}\right)}≜{\left({\text{X}}_{\left(n\right)}\right)}_{\left(:,{𝓘}_{n}\right)}\in {\mathbb{R}}^{D_{n}\times {\hat{R}}_{n}}$ as the matrix formed by these ${\hat{R}}_{n}$ columns, then (2) is restated as:

(5) $$𝓙({𝓘}_{n})={\left\|{\text{X}}_{\left(n\right)}-{\text{P}}_{{\left({\text{X}}_{\left(n\right)}\right)}_{{𝓘}_{n}}}{\text{X}}_{\left(n\right)}\right\|}_{\text{F}}^{2}$$

(6) $$=\;{\left\|{\text{X}}_{\left(n\right)}-{\left({\text{X}}_{\left(n\right)}\right)}_{{𝓘}_{n}}{\text{M}}_{{𝓘}_{n}}\right\|}_{\text{F}}^{2}$$

where ${\text{P}}_{{\left({\text{X}}_{\left(n\right)}\right)}_{{𝓘}_{n}}}={\left({\text{X}}_{\left(n\right)}\right)}_{{𝓘}_{n}}{{\left({\text{X}}_{\left(n\right)}\right)}_{{𝓘}_{n}}}^{†}\in {\mathbb{R}}^{D_{n}\times D_{n}}$ is the projection matrix corresponding to the column space of ${\left({\text{X}}_{\left(n\right)}\right)}_{{𝓘}_{n}}$, ${{\left({\text{X}}_{\left(n\right)}\right)}_{{𝓘}_{n}}}^{†}\in {\mathbb{R}}^{{\hat{R}}_{n}\times D_{n}}$ is the pseudoinverse of ${\left({\text{X}}_{\left(n\right)}\right)}_{{𝓘}_{n}}$, and ${\text{M}}_{{𝓘}_{n}}={{\left({\text{X}}_{\left(n\right)}\right)}_{{𝓘}_{n}}}^{†}{\text{X}}_{\left(n\right)}\in {\mathbb{R}}^{{\hat{R}}_{n}\times {\tilde{D}}_{n}}$ is the matrix mapping columns of ${\text{X}}_{\left(n\right)}$ onto ${\left({\text{X}}_{\left(n\right)}\right)}_{{𝓘}_{n}}$.

By denoting $𝓘=\{{𝓘}_{1},\;\ldots ,\;{𝓘}_{N}\}$ as the set of all N mode’s column index sets ${𝓘}_{n}$, for n=1,…,N, then we can represent the resulting decomposition’s cost in a manner similar to (1):

(7) $$𝓙(𝓘)={\left\|𝓧-𝓜\times_{1}{\left({\text{X}}_{\left(1\right)}\right)}_{{𝓘}_{1}}\times_{2}\ldots \times_{N}{\left({\text{X}}_{\left(N\right)}\right)}_{{𝓘}_{N}}\right\|}_{\text{F}}^{2}$$

where the core tensor is given by $𝓜=𝓧\times_{1}{{\left({\text{X}}_{\left(1\right)}\right)}_{{𝓘}_{1}}}^{†}\times_{2}\ldots \times_{N}{{\left({\text{X}}_{\left(N\right)}\right)}_{{𝓘}_{N}}}^{†}\in {\mathbb{R}}^{{\hat{R}}_{1}\times \ldots \times {\hat{R}}_{N}}$, and the factor matrices are given by the column subsets ${\left({\text{X}}_{\left(n\right)}\right)}_{{𝓘}_{n}}$.

This decomposition in (7) was first introduced in [80], referred to as “ApproxTensorSVD” in that paper. Later publications such as [83] refer to the algorithm as randomized sampling tucker CUR (RST-CUR). This decomposition is perhaps the most direct generalization of the matrix CUR to the tensor domain, as the decomposition takes the exact form of the matrix CUR when N=2. We refer to this decomposition as RST-CUR for the remainder of the paper.

The same advantages gained by matrix CUR for matrices carries over to RST-CUR for tensors, notably a low complexity way to approximate a tensor’s HOSVD. Additionally, as the factor matrices ${\left({\text{X}}_{\left(n\right)}\right)}_{{𝓘}_{n}}$ are fibers of the full tensor 𝓧, the factor matrices retain properties held by the original tensor, which can include sparsity, nonnegativity, etc.. These qualities in 𝓧 being retained in factor matrices ${\left({\text{X}}_{\left(n\right)}\right)}_{{𝓘}_{n}}$ may aid with the interpretability of the decomposition.

A key difference between column-based subset methods and row-based subset methods over ${\text{X}}_{\left(n\right)}$ is how differences in dimensions affect the subset selection process. As ${\text{X}}_{\left(n\right)}\in {\mathbb{R}}^{D_{n}\times {\tilde{D}}_{n}}$ is in general a very wide matrix with ${\tilde{D}}_{n}\;\gg \;D_{n}$, the massive number of columns leads deterministic column subset selection methods to be intractable, as their complexities are typically in the order of $𝓞({\tilde{D}}_{n}^{2})$ or more. Furthermore, even randomized methods typically only use a uniform distribution for sampling the columns, e.g. with norm sampling it may also be intractable to calculate the norm of all ${\tilde{D}}_{n}$ columns of ${\text{X}}_{\left(n\right)}$. This is a significant comparative disadvantage of the column-based methods such as RST-CUR, as uniform sampling of the columns may lead to significantly worse approximations for a given choice of ${\hat{R}}_{n}$. While column subset methods over ${\text{X}}_{\left(n\right)}$ are expensive, on the other hand, row-based subset methods are typically tractable due to the much smaller number of rows $D_{n}$, as we discuss in the next subsection.

### B. ROW-BASED SUBSET METHODS FOR TENSOR UNFOLDINGS

Row-based subset methods approximate a tensor unfolding ${\text{X}}_{\left(n\right)}$ using a subset of its rows. Aside from more advanced sampling methods being tractable over the rows than the columns of ${\text{X}}_{\left(n\right)}$, another advantage of row-based methods is the interpretability of their subsets. Because rows in ${\text{X}}_{\left(n\right)}$ are simply the elements of the nth mode, rows of ${\text{X}}_{\left(n\right)}$ are easier to interpret than the fiber columns of ${\text{X}}_{\left(n\right)}$.

For the nth mode unfolding ${\text{X}}_{\left(n\right)}$ of a tensor 𝓧, if we now denote ${𝓘}_{n}$ as an index set for some subset of ${\hat{R}}_{n}$ rows, and denote ${\left({\text{X}}_{\left(n\right)}\right)}_{\left({𝓘}_{n}\right)}≜{\left({\text{X}}_{\left(n\right)}\right)}_{\left({𝓘}_{n},:\right)}\in {\mathbb{R}}^{{\hat{R}}_{n}\times {\tilde{D}}_{n}}$ as the matrix formed by these ${\hat{R}}_{n}$ rows, then (2) is restated as:

(8) $$𝓙({𝓘}_{n})={\left\|{\text{X}}_{\left(n\right)}-{\text{X}}_{\left(n\right)}{\text{P}}_{{\left({\text{X}}_{\left(n\right)}\right)}_{{𝓘}_{n}}}\right\|}_{\text{F}}^{2}$$

(9) $$=\;{\left\|{\text{X}}_{\left(n\right)}-{\text{M}}_{{𝓘}_{n}}{\left({\text{X}}_{\left(n\right)}\right)}_{{𝓘}_{n}}\right\|}_{\text{F}}^{2}$$

where ${\text{P}}_{{\left({\text{X}}_{\left(n\right)}\right)}_{{𝓘}_{n}}}\in {\mathbb{R}}^{T_{n}\times {\tilde{D}}_{n}}$ is the projection matrix corresponding for the row space of ${\left({\text{X}}_{\left(n\right)}\right)}_{{𝓘}_{n}}$, and ${\text{M}}_{{𝓘}_{n}}\in {\mathbb{R}}^{D_{n}\times {\hat{R}}_{n}}$ is the nth mode’s mapping matrix, which maps rows of X onto the span of ${\left({\text{X}}_{\left(n\right)}\right)}_{{𝓘}_{n}}$, and is given by:

(10) $${\text{M}}_{{𝓘}_{n}}={\text{X}}_{\left(n\right)}{\left({\text{X}}_{\left(n\right)}\right)}_{{𝓘}_{n}}^{⊤}{\left({\left({\text{X}}_{\left(n\right)}\right)}_{\left({𝓘}_{n}\right)}{\left({\text{X}}_{\left(n\right)}\right)}_{{𝓘}_{n}}^{⊤}\right)}^{-1}$$

By denoting $𝓘=\{{𝓘}_{1},\;\ldots ,\;{𝓘}_{N}\}$ as the set of all N modes’ row index sets ${𝓘}_{n}$, for n=1,…,N, then we can represent the resulting decomposition’s cost in a form similar to (1):

(11) $$𝓙(𝓘)={\left\|𝓧-{𝓧}_{𝓘}\times_{1}{\text{M}}_{{𝓘}_{1}}\times_{2}\ldots \times_{N}{\text{M}}_{{𝓘}_{N}}\right\|}_{\text{F}}^{2}$$

where the core tensor ${𝓧}_{𝓘}={\left(𝓧\right)}_{\left({𝓘}_{1},\;\ldots ,\;{𝓘}_{N}\right)}\in {\mathbb{R}}^{{\hat{R}}_{1}\times \ldots \times {\hat{R}}_{N}}$ is a subtensor of 𝓧 over the index sets ${𝓘}_{n}$, and the factor matrices are the N mapping matrices ${\text{M}}_{{𝓘}_{N}}$, for n=1,…,N.

The characteristic difference between the decomposition ${𝓧}_{𝓘}\times_{1}{\text{M}}_{{𝓘}_{1}}\times_{2}\ldots \times_{N}{\text{M}}_{{𝓘}_{N}}$ in (11), and the decomposition $𝓜\times_{1}{\left({\text{X}}_{\left(1\right)}\right)}_{{𝓘}_{1}}\times_{2}\ldots \times_{N}{\left({\text{X}}_{\left(N\right)}\right)}_{{𝓘}_{N}}$ in (7), is how elements of the tensor 𝓧 manifest as elements in the decomposition, relative to a tensor generalization of (3). In (7), elements of 𝓧 manifest as fibers in the *factor matrices ${\left({\text{X}}_{\left(n\right)}\right)}_{{𝓘}_{n}}$*, and the core tensor 𝓜 can be considered a tensor generalization of the *mapping matrix*. Where in (11), the opposite occurs: elements of 𝓧 manifest as the *core tensor ${𝓧}_{𝓘}$*, and the *factor matrices ${\text{M}}_{{𝓘}_{N}}$* are the N modes’ mapping matrices. Thus with (11), the core tensor is the element of the decomposition that retains properties of the original tensor, which may yield more useful decompositions depending on the application.

Various tensor decompositions take the form of the decomposition ${𝓧}_{𝓘}\times_{1}{\text{M}}_{{𝓘}_{1}}\times_{2}\ldots \times_{N}{\text{M}}_{{𝓘}_{N}}$ in (11). This decomposition was first introduced in [79] shortly before the introduction of the RST-CUR decomposition. Later works such as [82] have provided significant understandings to the error guarantees of this decomposition, and have referred to it by the name “Chidori CUR” decomposition.

A key feature of the Chidori CUR is that the subset indices ${𝓘}_{N}$ are chosen prior to the decomposition, and that the mapping matrices ${\text{M}}_{{𝓘}_{N}}$ are calculated only over those fibers of ${𝓧}_{\left(n\right)}$ that correspond to the subset indices ${𝓘}_{N}$ of all N−1 other modes. In other words, in calculation of ${\text{M}}_{{𝓘}_{N}}$ in (10), the matrix ${\text{X}}_{\left(n\right)}$ is the unfolding of the nth mode “Chodiri Beam” ${\left(𝓧\right)}_{\left({𝓘}_{1},\ldots ,{𝓘}_{n-1},:,{𝓘}_{n+1},\ldots ,{𝓘}_{N}\right)}\in {\mathbb{R}}^{{\hat{R}}_{1}\times \ldots \times {\hat{R}}_{n-1}\times D_{1}\times {\hat{R}}_{n+1}\times \ldots \times {\hat{R}}_{n}}$, and ${\left({\text{X}}_{\left(n\right)}\right)}_{{𝓘}_{n}}$ is the unfolding of the core tensor ${𝓧}_{𝓘}$ (a subtensor of the nth Chidori Beam). Because the ${\text{M}}_{{𝓘}_{N}}$ are calculated over only the Chidori Beams, the decomposition only requires access to the Chidori beams and is thus independent from all other elements in the tensor. This reliance on only a small subset of the tensor to perform the decomposition results in one of the most computationally efficient tensor decompositions. At the same time, however, independence of the decomposition from elements outside the Chidori beams may result in a worse factorization than other decompositions, particularly when the subsets of the core tensor ${𝓧}_{𝓘}$ are not well-representative of the rest of 𝓧, or if 𝓧 is otherwise heavily heterogeneous in nature.

A similar decomposition was later introduced in [81], and can be considered a generalization of the Chidori CUR where the unfolding fibers in ${\text{X}}_{\left(n\right)}$ and ${\left({\text{X}}_{\left(n\right)}\right)}_{{𝓘}_{n}}$ are not restricted to those ${𝓘}_{N}$ of the N−1 other modes, but can be any random corresponding subset of fibers from ${\text{X}}_{\left(n\right)}$ and ${\left({\text{X}}_{\left(n\right)}\right)}_{{𝓘}_{n}}$ over the entire tensor. This decomposition was also later studied in [82] and has been called the “Fiber CUR” decomposition. As the Fiber CUR allows access to any random subset of fibers of ${\text{X}}_{\left(n\right)}$ and ${\left({\text{X}}_{\left(n\right)}\right)}_{{𝓘}_{n}}$ for calculating mapping matrices ${\text{M}}_{{𝓘}_{N}}$, its decomposition may be more robust to poorly chosen subsets of the data. However, as column fibers in Fiber CUR are typically uniformly sampled, this may also lead the Fiber CUR to exhibit considerably higher variation in the quality of the estimated ${\text{M}}_{{𝓘}_{N}}$, which often leads to worse decompositions than those provided by Chidori CUR.

As described in Section III-A, the massive numbers of columns in ${\text{X}}_{\left(n\right)}$ make column selection methods intractable, and thus typically only rely on uniform sampling to select the columns. However for row-based methods such as Chidori CUR and Fiber CUR, the much smaller number of rows $D_{n}\;\ll \;{\tilde{D}}_{n}$ allow for more sophisticated sampling methods such as norm sampling. When norm sampling is applied, index sets ${𝓘}_{N}$ are selected according to the norms of elements in the original tensor, e.g. ${\left({\text{X}}_{\left(n\right)}\right)}_{\left(i,:\right)}$ which when vectorized is of dimension ${\tilde{D}}_{n}$. These sampling schemes require N passes over the tensor to construct the N index sets, and thus can still be of considerable expense. Perhaps as a result of this, row-based subset methods for tensors have exclusively used random subset methods such as uniform and norm sampling to obtain subsets of the tensor, and thus deterministic subset methods have not been explored.

Building on the ideas presented in previous sections, in the next section we introduce a new way of performing a subset-based Tucker decomposition that provides a good balance between efficiency and approximation quality, by exploiting weighted subsets of the data called *coresets*.

## IV. TENSOR CORESET DECOMPOSITION

In this section, we introduce a method for the Tucker decomposition that operates by selecting *coresets*: weighted subsets of the data. As we later explain, these weighted subsets can provide a better approximation to the tensor 𝓧 by effectively better approximating the HOSVD’s principal component tensor 𝓖. We later motivate additional differences vs. the previously discussed methods, such as a sequentially truncated coresets approach analogous to ST-HOSVD, and the ability to represent symmetry in the tensor over multiple modes. Furthermore, instead of exclusively selecting subsets randomly for greater efficiency, we also motivate ability to select subsets deterministically, for better approximation quality and for feature selection.

### A. SUBSET DISCREPANCY–A MEASURE OF “REPRESENTATIVENESS”

To motivate weighted subsets within a tensor, we first refer back to the per-mode approximation error provided for row-based subsets in (9).


$$𝓙({𝓘}_{n})={\left\|{\text{X}}_{\left(n\right)}-{\text{M}}_{{𝓘}_{n}}{\left({\text{X}}_{\left(n\right)}\right)}_{{𝓘}_{n}}\right\|}_{\text{F}}^{2}$$

Denoting the SVD of ${\text{X}}_{\left(n\right)}$ by ${\text{X}}_{\left(n\right)}={\text{U}}_{{\text{X}}_{\left(n\right)}}\sum_{{\text{X}}_{\left(n\right)}}{\text{V}}_{{\text{X}}_{\left(n\right)}}^{⊤}$, the approximation error of ${\text{M}}_{{𝓘}_{n}}{\left({\text{X}}_{\left(n\right)}\right)}_{{𝓘}_{n}}\in {\mathbb{R}}^{D_{n}\times {\tilde{D}}_{n}}$ depends on how well the subset ${\left({\text{X}}_{\left(n\right)}\right)}_{{𝓘}_{n}}$ can approximate the row-space of ${\text{X}}_{\left(n\right)}$, specifically in terms of approximating its right principal components $\sum_{{\text{X}}_{\left(n\right)}}{\text{V}}_{{\text{X}}_{\left(n\right)}}^{⊤}$, which in the tensor domain is represented by the HOSVD core tensor 𝓖 These PCs are analytically given by the eigenvectors ${\text{V}}_{{\text{X}}_{\left(n\right)}}$ and corresponding eigenvalues $\sum_{{\text{X}}_{\left(n\right)}}$ of the quadratic form ${\text{X}}_{\text{(}n\text{)}}^{⊤}{\text{X}}_{\left(n\right)}\in {\mathbb{R}}^{T_{n}\times {\tilde{D}}_{n}}$, and thus can be approximated from ${\left({\text{X}}_{\left(n\right)}\right)}_{{𝓘}_{n}}$ via the corresponding form ${\left({\text{X}}_{\left(n\right)}\right)}_{{𝓘}_{n}}^{⊤}{\left({\text{X}}_{\left(n\right)}\right)}_{{𝓘}_{n}}\in {\mathbb{R}}^{T_{n}\times {\tilde{D}}_{n}}$. As a result, an implicit distance between the PCs of ${\text{X}}_{\left(n\right)}$ and ${\left({\text{X}}_{\left(n\right)}\right)}_{{𝓘}_{n}}$ is given by the distance between the quadratic forms:

(12) $$𝓡({𝓘}_{n})={\left\|{\left({\text{X}}_{\left(n\right)}\right)}_{{𝓘}_{s}}^{⊤}{\left({\text{X}}_{\left(n\right)}\right)}_{{𝓘}_{s}}-{\text{X}}_{\text{(}n\text{)}}^{⊤}{\text{X}}_{\left(n\right)}\right\|}_{\text{F}}^{2}$$

This can be understood as a nonparametric measure of *discrepancy* [90], [91], [92], [93], [94], [95] between the full set ${\text{X}}_{\left(n\right)}$ and the subset ${\left({\text{X}}_{\left(n\right)}\right)}_{{𝓘}_{n}}$, analogous to the maximum mean discrepancy [92], [93], [94] for a particular realization of distributional “embeddings” of the elements in the set. Specifically for some ith element in the nth node, given by ${\left({\text{X}}_{\left(n\right)}\right)}_{\left(i,:\right)}$, its corresponding embedding in this discrepancy is given by ${\left({\text{X}}_{\left(n\right)}\right)}_{\left(i,:\right)}^{⊤}{\left({\text{X}}_{\left(n\right)}\right)}_{\left(i,:\right)}\in {\mathbb{R}}^{{\tilde{D}}_{n}\times {\tilde{D}}_{n}}$, and the nth mode’s “full mode embedding” ${\text{X}}_{\text{(}n\text{)}}^{⊤}{\text{X}}_{\left(n\right)}$ is given by the sum ${\text{X}}_{\text{(}n\text{)}}^{⊤}{\text{X}}_{\left(n\right)}=\sum_{i=1}^{D_{n}}{\left({\text{X}}_{\left(n\right)}\right)}_{\left(i,:\right)}^{⊤}{\left({\text{X}}_{\left(n\right)}\right)}_{\left(i,:\right)}$, which we seek to best approximate via the subset’s embedding ${\left({\text{X}}_{\left(n\right)}\right)}_{{𝓘}_{n}}^{⊤}{\left({\text{X}}_{\left(n\right)}\right)}_{{𝓘}_{n}}$.

### B. CORESETS–WEIGHTED SUBSETS

A subset’s discrepancy can be further decreased by *weighting* the subset: assigning individual weights $w_{n}^{\left[i\right]}$ to each ith element in the subset. Utilizing these weighted subsets, called *coresets*, the discrepancy measure is given by:

(13) $$𝓡({𝓘}_{n},{\text{w}}_{n})={\left\|\sum_{i\in {𝓘}_{n}}w_{n}^{\left[i\right]}{\left({\text{X}}_{\left(n\right)}\right)}_{\left(i,:\right)}^{⊤}{\left({\text{X}}_{\left(n\right)}\right)}_{\left(i,:\right)}-{\text{B}}_{n}\right\|}_{\text{F}}^{2}$$

where ${\text{w}}_{n}=[w_{n}^{\left[1\right]},\ldots ,w_{n}^{\left[{\hat{R}}_{n}\right]}]\in {\mathbb{R}}^{{\hat{R}}_{n}}$ is the set of ${\hat{R}}_{n}$ coreset weights corresponding to each element in ${\left({\text{X}}_{\left(n\right)}\right)}_{{𝓘}_{n}}\in {\mathbb{R}}^{{\hat{R}}_{n}\times {\tilde{D}}_{n}}$, and ${\text{B}}_{n}≜{\text{X}}_{\text{(}n\text{)}}^{⊤}{\text{X}}_{\left(n\right)}\in {\mathbb{R}}^{{\tilde{D}}_{n}\times {\tilde{D}}_{n}}$ is the nth modes full mode embedding (a fixed quantity).

An important point to acknowledge here is that in (13), the weights $w_{n}^{\left[i\right]}$ are applied to the element *embeddings* ${\left({\text{X}}_{\left(n\right)}\right)}_{\left(i,:\right)}^{⊤}{\left({\text{X}}_{\left(n\right)}\right)}_{\left(i,:\right)}$, and not the elements themselves ${\left({\text{X}}_{\left(n\right)}\right)}_{\left(i,:\right)}$. Instead, it follows that the elements receive the square root of the weights ${\left(w_{n}^{\left[i\right]}\right)}^{\frac{1}{2}}$:

$$\begin{array}{l} w_{n}^{\left[i\right]}({\left({\text{X}}_{\left(n\right)}\right)}_{\left(i,:\right)}^{⊤}{\left({\text{X}}_{\left(n\right)}\right)}_{\left(i,:\right)})\\ ={\left({\left(w_{n}^{\left[i\right]}\right)}^{\frac{1}{2}}{\left({\text{X}}_{\left(n\right)}\right)}_{\left(i,:\right)}\right)}^{⊤}({\left(w_{n}^{\left[i\right]}\right)}^{\frac{1}{2}}{\left({\text{X}}_{\left(n\right)}\right)}_{\left(i,:\right)}) \end{array}$$

This necessitates us to later specify nonnegative weights $w_{n}^{\left[i\right]}\geq 0$ in order for ${\left(w_{n}^{\left[i\right]}\right)}^{\frac{1}{2}}$ to be real.

We now discuss the procedure for selecting weights ${\text{w}}_{n}$ that minimize the discrepancy (13). For simplicity, for now we assume that we have a particular realization of the subset ${𝓘}_{N}$, which is selected either randomly or deterministically (as we explain in the next subsection). With ${𝓘}_{N}$ fixed and discrepancy as only a function of the weights ${\text{w}}_{n}$, we can equivalently write (13) in a form where all ${\tilde{D}}_{n}\times {\tilde{D}}_{n}$ matrices are instead given as ${\tilde{D}}_{n}^{2}\times 1$ vectors:

(14) $$𝓡({\text{w}}_{n})={\left\|\sum_{i\in {𝓘}_{n}}w_{n}^{\left[i\right]}{\text{a}}_{n}^{\left[i\right]}-{\text{b}}_{n}\right\|}_{2}^{2}\;={\left\|{\text{A}}_{n}{\text{w}}_{n}-{\text{b}}_{n}\right\|}_{2}^{2}$$

where we define ${\text{a}}_{n}^{\left[i\right]}=\text{vec}({\left({\text{X}}_{\left(n\right)}\right)}_{\left(i,:\right)}^{⊤}{\left({\text{X}}_{\left(n\right)}\right)}_{\left(i,:\right)})\in {\mathbb{R}}^{T^{2}}$ as the vectorization of the ith element’s embedding, ${\text{A}}_{n}=[{\text{a}}_{n}^{\left[1\right]},\ldots ,{\text{a}}_{n}^{\left[{\hat{R}}_{n}\right]}]\in {\mathbb{R}}^{{\tilde{D}}_{n}^{2}\times {\hat{R}}_{n}}$ as the horizontal concatenation of the ${\text{a}}_{n}^{\left[i\right]}$, and ${\text{b}}_{n}=\text{vec}({\text{B}}_{n})\in {\mathbb{R}}^{{\tilde{D}}_{n}^{2}}$ as the vectorization of the full mode embedding.

This is a least squares problem Anwn−bnwnargmin22 for which the ordinary least squares (OLS) solution is ${\text{w}}_{n}={\left({\text{A}}_{n}^{⊤}{\text{A}}_{n}\right)}^{-1}{\text{A}}_{n}^{⊤}{\text{b}}_{n}$. However as noted previously, we also require that the weights $w_{n}^{\left[i\right]}$ be nonnegative in order for the square root of weights (applied to the elements themselves) to be real. Therefore, we use a NNLS algorithm [110] to solve for ${\text{w}}_{n}$. This is an efficient algorithm that does not explicitly form the ${\tilde{D}}_{n}\times {\tilde{D}}_{n}$ embeddings, instead only requiring the kernels between embeddings which are significantly easier to calculate. We define the kernel between the embeddings of ${\left({\text{X}}_{\left(n\right)}\right)}_{\left(i,:\right)}$ and ${\left({\text{X}}_{\left(n\right)}\right)}_{\left(j,:\right)}$ as:

(15) $$\begin{array}{cc} \text{k}(i_{n},j_{n})=<{\text{a}}_{n}^{\left[i\right]},{\text{a}}_{n}^{\left[j\right]}> & ={\left({\left({\text{X}}_{\left(n\right)}\right)}_{\left(i,:\right)}{\left({\text{X}}_{\left(n\right)}\right)}_{\left(j,:\right)}^{⊤}\right)}^{2} \end{array}$$

The two quantities required by the algorithm are kernels $({\text{A}}_{n}^{⊤}{\text{A}}_{n})\in {\mathbb{R}}^{{\hat{R}}_{n}\times {\hat{R}}_{n}}$ and ${\text{A}}_{n}^{⊤}{\text{b}}_{n}\in {\mathbb{R}}^{{\hat{R}}_{n}}$. The matrix $\left({\text{A}}_{n}^{⊤}{\text{A}}_{n}\right)$ provides all pairwise kernels within the ${𝓘}_{N}$ subset, and is equal to ${\left({\left({\text{X}}_{\left(n\right)}\right)}_{\left({𝓘}_{n},:\right)}{\left({\text{X}}_{\left(n\right)}\right)}_{\left({𝓘}_{n},:\right)}^{⊤}\right)}^{\circ 2}\in {\mathbb{R}}^{{\hat{R}}_{n}\times {\hat{R}}_{n}}$, where ${\left(\cdot \right)}^{\circ 2}$ denotes the Hadamard power (here, elementwise squaring). The vector ${\text{A}}_{n}^{⊤}{\text{b}}_{n}$ provides kernels with each element in the subset with the full mode embedding, equal to ${\left({\left({\text{X}}_{\left(n\right)}\right)}_{\left({𝓘}_{n},:\right)}{\text{X}}_{\left(n\right)}^{⊤}\right)}^{\circ 2}\text{1}\in {\mathbb{R}}^{{\hat{R}}_{n}}$, where $\text{1}\in {\mathbb{R}}^{D_{n}}$ is the vector of 1s. For further efficiency, we initialize the NNLS algorithm with the mapping of the OLS solution ${\left({\text{A}}_{n}^{⊤}{\text{A}}_{n}\right)}^{-1}{\text{A}}_{n}^{⊤}{\text{b}}_{n}$ to its nearest nonnegative vector. In our experience, we often observe that the OLS solution is already nonnegative and thus exactly minimizes (14) without requiring the NNLS algorithm.

Having provided the means to optimize the weights ${\text{w}}_{n}$, in the next section we discuss ways of selecting the subsets.

### C. SUBSET SELECTION–RANDOMIZED OR DETERMINISTIC

As our method is a row-based subset method over ${\text{X}}_{\left(n\right)}$, we can consider more advanced means of selecting subsets ${\left({\text{X}}_{\left(n\right)}\right)}_{\left({𝓘}_{n},:\right)}$ than uniform sampling of rows. We use different strategies if we seek random subsets, prioritizing computational efficiency over approximation quality, or deterministic subsets, prioritizing approximation quality in addition to the utility of feature selection.

For random subsets, we use norm sampling as done with previously mentioned methods. While we unfortunately do not provide an approximation bound for random subsets using the NNLS weights discussed previously, intuitively these weights should yield a discrepancy that is less than or equal to that provided by the normalized weights discussed in (4), as those weights are not explicitly optimizing over the discrepancy whereas the NNLS weights are. Therefore, we expect an error superior or equal to that of (4)’s weights. As we show in the next section, it is inexpensive to calculate the NNLS weights since the kernel quantities are required anyways to calculate the nth mode’s mapping matrix ${\text{M}}_{{𝓘}_{n}}$. Selection of the random subset along with calculating the weights has complexity of $𝓞(({\hat{R}}_{n}+1){\tilde{D}}_{n}D_{n}+{\hat{R}}_{n}^{3})$, which is linear in $D_{n}$.

For deterministic subsets, we retain the use of greedy methods in the interest of balancing approximation quality with computational efficiency. As discussed in Section II-C, greedy methods significantly outperform the error bounds of randomized methods and lead to subsets that rapidly converge to the properties of the full set. We specifically utilize the weighted kernel herding (WKH) method [95] which allows us to simultaneously and efficiently solve for the subset indices ${𝓘}_{N}$ and weights ${\text{w}}_{n}$. Like the NNLS algorithm, the WKH algorithm is made more efficient by only requiring kernels to operate. It uses ${\text{X}}_{\left(n\right)}{\text{X}}_{\left(n\right)}^{⊤}\in {\mathbb{R}}^{D_{n}\times D_{n}}$, the matrix of pairwise kernels between all elements in the nth mode, and has complexity $𝓞({\tilde{D}}_{n}D_{n}^{2}+{\hat{R}}_{n}^{3}D_{n})$, which is quadratic in $D_{n}$.

In the next section, we introduce our tensor decomposition method as a sequentially truncated variation of the row-based subset model in (11), where we sequentially replace the tensor with a coreset of itself.

### D. TENSOR DECOMPOSITION VIA SEQUENTIALLY TRUNCATED CORESETS

We now motivate our method for performing a coreset-based Tensor decomposition. We first revisit points mentioned in Section III-B. specifically discussing the advantages and disadvantages of the Chidori CUR decomposition. As we note previously, the Chidori CUR Decomposition is efficient because it only requires processing small subsets of the tensor – the “Chidori beams” – in order to calculate the mode’s mapping matrices ${\text{M}}_{{𝓘}_{n}}$. However, this may also lead to a significantly worse approximation quality for the decomposition, in the event that the randomly chosen subsets are not well representative of the entire tensor X, or otherwise for decomposing tensors that are highly heterogeneous in nature. When approximation quality is a priority for both randomized and deterministic methods, it may be more prudent to use a method that does pass over all elements of the tensor, but preferably only once if computational efficiency is also a priority. These decompositions can provide significantly more representative subsets of the data while still maintaining excellent computational efficiency.

With this focus in mind, in order to provide a good balance between approximation error and efficiency, we instead consider a method inspired by ST-HOSVD that utilizes *sequentially truncated coresets* to perform the decomposition. Like ST-HOSVD, for each nth mode of the tensor, we would learn the mapping matrix ${\text{M}}_{{𝓘}_{n}}$ and then replace the tensor with a truncated tensor, thus significantly decreasing the complexity of calculating ${\text{M}}_{{𝓘}_{n}}$ for all remaining modes. However, whereas ST-HOSVD replaces the tensor with the PCs of the mode, we instead replace the tensor with the nth mode’s coreset. These methods are closely connected by the fact that the coresets are trying to best preserve the PCs of the tensor, as evidence by the discrepancy cost in (12) and (13).

**FIGURE 1.**
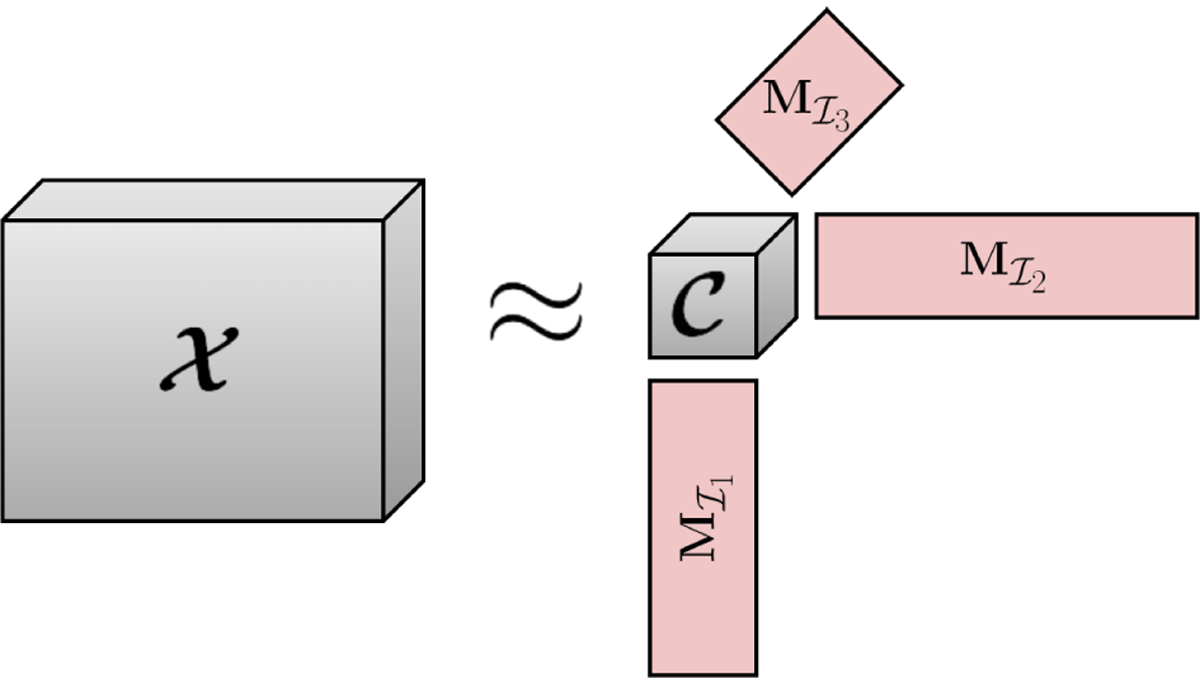
Visualization of the tensor coreset decomposition (TCD) applied to a 3rd-order tensor 𝓧. The core tensor 𝓒 is a weighted subtensor (coreset) of 𝓧.

We now discuss details of our method’s implementation to assist understanding the pseudocode provided in Algorithm 4. The factorization of the nth mode is initialized by selecting a subset ${𝓘}_{N}$, which as we mentioned in Section VI.C is in general $𝓞\left(D_{n}\right)$ for random subsets or $𝓞\left(D_{n}^{2}\right)$ for deterministic subsets. We then compute the pairwise inner products between the subset and the full set, given by the matrix ${\text{P}}_{{𝓘}_{n}}={\left({\text{X}}_{\left(n\right)}\right)}_{{𝓘}_{n}}{\text{X}}_{\left(n\right)}^{Τ})\in {\mathbb{R}}^{{\hat{R}}_{n}\times D_{n}}$, within which the submatrix ${\text{P}}_{\left({𝓘}_{n},{𝓘}_{n}\right)}={\left({\text{P}}_{{𝓘}_{n}}\right)}_{\left(:,{𝓘}_{n}\right)}\in {\mathbb{R}}^{{\hat{R}}_{n}\times {\hat{R}}_{n}}$ provides the pairwise inner products within the subset. With both of these matrices, we can obtain the kernels of embeddings ${\text{P}}_{\left({𝓘}_{n},{𝓘}_{n}\right)}^{\circ 2}\in {\mathbb{R}}^{{\hat{R}}_{n}\times {\hat{R}}_{n}}$ and ${\text{P}}_{\left({𝓘}_{n}\right)}^{\circ 2}{\text{1}}_{D_{n}}\in {\mathbb{R}}^{{\hat{R}}_{n}}$ to perform NNLS and learn the coreset weights ${\text{w}}_{n}\in {\mathbb{R}}^{{\hat{R}}_{n}}$. Arranging ${\text{w}}_{n}$ in the diagonal matrix ${\text{W}}_{n}\in {\mathbb{R}}^{{\hat{R}}_{n}\times {\hat{R}}_{n}}$, the approximation in (9) given by ${\text{M}}_{{𝓘}_{n}}{\left({\text{X}}_{\left(n\right)}\right)}_{{𝓘}_{n}}$ can be weighted via ${\text{M}}_{{𝓘}_{n}}{\text{W}}_{n}^{-1}{\text{W}}_{n}{\left({\text{X}}_{\left(n\right)}\right)}_{{𝓘}_{n}}$, in which case the mapping matrix (accounting for weights) is given by ${\text{M}}_{{𝓘}_{n}}={\text{P}}_{{𝓘}_{n}}^{⊤}{\text{P}}_{\left({𝓘}_{n},{𝓘}_{n}\right)}^{-1}{\text{W}}_{n}^{-1}\in {\mathbb{R}}^{D_{n}\times {\hat{R}}_{n}}$, and the weighted coreset is given by ${\text{W}}_{n}{\left({\text{X}}_{\left(n\right)}\right)}_{{𝓘}_{n}}$. Thus with the weights calculated, we compute the mapping matrix ${\text{M}}_{{𝓘}_{n}}$, then truncate the nth mode by replacing it with the coreset ${\text{W}}_{n}{\left({\text{X}}_{\left(n\right)}\right)}_{{𝓘}_{n}}$, and finally un-matricize the tensor so that the entire process can be repeated for the remaining modes. Fig. 1 visualizes the tensor coreset decomposition (TCD).

After truncating over all modes, the resulting coreset core tensor $𝓒={𝓧}_{𝓘}\times_{1}{\text{W}}_{1}\times_{2}\ldots \times_{N}{\text{W}}_{N}$ is a subtensor ${𝓧}_{𝓘}$ weighted on each nth mode by weight matrix ${\text{W}}_{n}$, and serves as a compressed form of 𝓧 analogous to the principal component tensor 𝓖 from HOSVD. The weights ${\text{W}}_{n}$ are a key differentiator from other methods like the Chidori CUR decomposition, and the use of ${\text{W}}_{n}$ within this sequentially method can allow for an excellent approximation to the HOSVD core tensor 𝓖, and by extension, the tensor 𝓧.

The method described in Algorithm 4 assumes that 𝓧 is an asymmetric tensor, and does not preserve symmetry in the decomposition if 𝓧 is symmetric across several modes. To retain symmetry in the decomposition in the event that 𝓧 is symmetric, we simply compute only one factor matrix ${\text{M}}_{{𝓘}_{n}}$ for one of the symmetric modes n, and reuse ${\text{M}}_{{𝓘}_{n}}$ for all other modes symmetric to n, while truncating those other modes the same way ${\text{X}}_{\left(n\right)}\to {\text{W}}_{n}{\left({\text{X}}_{\left(n\right)}\right)}_{{𝓘}_{n}}$.

In the next subsection, we compare our so called Tensor Coreset Decomposition (TCD) to the Chidori CUR decomposition from a computational complexity standpoint.

### E. COMPUTATIONAL COMPLEXITY OF TCD

In this section, we discuss the complexities of TCD with random (norm sampled) or deterministic (WKH) subsets, compared to Chidori CUR with random (norm sampled) subsets. We retain notations such as ${\tilde{D}}_{n}^{\left(n\right)}=({\prod_{m=1}^{n-1}\hat{R}}_{m})({\prod_{m=n+1}^{N}D}_{m})$ for sequentially truncated methods like ST-HOSVD and TCD. For simplicity, we assume that the modes truncated with these methods are in the order n=1,…,N.

We first discuss complexity of Chidori CUR decomposition with random (norm sampled) subsets. The majority of complexity is in calculation of the norms of elements across the N modes of the original tensor 𝓧, each mode of complexity $𝓞(D_{n}{\tilde{D}}_{n})$. These are then followed by the significantly cheaper calculations of the mapping matrices per each Chidori Beam, of complexity $𝓞({\hat{R}}_{n}D_{n}{\hat{T}}_{n}+{\hat{R}}_{n}^{3}+{\hat{R}}_{n}^{2}D_{n})$, where we denote ${\hat{T}}_{n}=\prod_{\begin{array}{l} m=1\\ m\neq n \end{array}}^{N}{\hat{R}}_{m}$. The total complexity is thus $𝓞(\sum_{n=1}^{N}\left(D_{n}{\tilde{D}}_{n}+{\hat{R}}_{n}D_{n}{\hat{T}}_{n}+{\hat{R}}_{n}^{3}+{\hat{R}}_{n}^{2}D_{n}\right))$.


$$\begin{array}{l} \bar{\underline{\text{Algorithm}\;\text{4}\;\text{Tensor}\;\text{Coreset}\;\text{Decomposition}\;\left(\text{TCD}\right)}}\\ \underline{\begin{array}{l} \text{Input:}𝓧\in {\mathbb{R}}^{D_{1}\times \ldots \times D_{N}}(N\text{-mode}\;\text{tensor}),\\ \;\;\;[{\hat{R}}_{1},\ldots ,{\hat{R}}_{N}](\text{subset}\;\text{sizes}\;\text{per}\;\text{mode)}\\ \text{Output:}𝓧\approx 𝓒\times_{1}{\text{M}}_{{𝓘}_{1}}\times \ldots \times_{N}{\text{M}}_{{𝓘}_{N}},\text{where}\\ \;\;\;𝓒\in {\mathbb{R}}^{{\hat{R}}_{1}\times \ldots \times {\hat{R}}_{N}}\;\text{(coreset}\;\text{tensor),}\\ \;\;\;\{{\text{M}}_{{𝓘}_{1}},\ldots ,{\text{M}}_{{𝓘}_{N}}\}\;\text{(mapping matrices per mode}\\ \;\;\;\;\;\;-\text{like factor matrices}),\\ \;\;\;𝓘=\{{𝓘}_{1},\;\ldots ,\;{𝓘}_{N}\}\;\text{(subset}\;\text{index}\;\text{sets}\;\text{per}\;\text{mode)}\\ \;\text{for}\;\text{each}\;\text{mode}\;n\;\text{=1}\;\text{:}N\\ \;\;\text{unfold}\;\text{(matricize)}\;\text{tensor}\;\text{w.r.t.}\;n\text{th}\;\text{mode}\\ \;\;\;𝓧\to {\text{X}}_{\left(n\right)}\in {\mathbb{R}}^{D_{n}\times {\tilde{D}}_{n}},\text{with}\;{\tilde{D}}_{n}=\prod_{\begin{array}{l} m=1\\ m\neq n \end{array}}^{N}D_{m}\\ \;\;\text{select}\;\text{subset}\;\text{indices}\;{𝓘}_{n}\;\text{for}\;\text{some}\;{\hat{R}}_{n}\;\text{rows}\;\text{of}\;{\text{X}}_{\left(n\right)},\;\\ \;\;\;\text{either}\;\text{randomly}\;\text{(using}\;\text{e.g.}\;\text{norm}\;\text{sampling),}\\ \;\;\;\text{or}\;\text{deterministically}\;\text{(using}\;\text{e.g.}\;\text{greedy}\;\text{WKH).}\\ \;\;\text{compute inner products of full mode with subset:}\\ \;\;\;{\text{P}}_{{𝓘}_{n}}={\left(X_{\left(n\right)}\right)}_{{𝓘}_{n}}X_{\left(n\right)}^{⊤}\in {\mathbb{R}}^{{\hat{R}}_{n}\times D_{n}},\\ \;\;\;\text{also}\;\text{within}\;{\text{P}}_{{𝓘}_{n}}\;\text{the}\;\text{submatrix}\;{\text{P}}_{\left({𝓘}_{n},{𝓘}_{n}\right)}\in {\mathbb{R}}^{{\hat{R}}_{n}\times {\hat{R}}_{n}}\;\\ \;\;\text{using}\;\text{the}\;\text{kernels}\;{\text{P}}_{\left({𝓘}_{n},{𝓘}_{n}\right)}^{\circ 2}\in {\mathbb{R}}^{{\hat{R}}_{n}\times {\hat{R}}_{n}}\\ \;\;\;\text{and}\;{\text{P}}_{\left({𝓘}_{n}\right)}^{\circ 2}{\text{1}}_{D_{n}}\in {\mathbb{R}}^{{\hat{R}}_{n}},\text{perform}\;\text{kernel}\;\text{NNLS}\\ \;\;\;\text{to}\;\text{learn}\;\text{coreset}\;\text{weights}\;\text{w}\in {\mathbb{R}}^{{\hat{R}}_{n}}.\\ \;\;\text{arrange}\;\text{weights}\;\text{into}\;\text{diagonal}\;\text{matrix}\;{\text{W}}_{n}=\text{diag(w)}\\ \;\;\text{compute}\;\text{and}\;\text{store}\;n\text{th}\;\text{mode's}\;\text{mapping}\;\text{matrix:}\\ \;\;\;{\text{M}}_{{𝓘}_{n}}={\text{P}}_{{𝓘}_{n}}^{⊤}{\text{P}}_{\left({𝓘}_{n},{𝓘}_{n}\right)}^{⊤}{\text{W}}_{n}^{-1}\in {\mathbb{R}}^{D_{n}\times {\hat{R}}_{n}}\\ \;\;\text{replace}\;\text{unfolded}\;\text{tensor}\;\text{with}\;\text{weighted}\;\text{coreset}\\ \;\;\;\text{X}\mathrm{(n)}\to {\text{W}}_{n}{\left(\text{X}\left(\text{n}\right)\right)}_{{𝓘}_{n}}\in {\mathbb{R}}^{{\hat{R}}_{n}\times {\tilde{D}}_{n}}\\ \;\;\text{replace}\;n\text{th}\;\text{dimension}\;D_{n}\to {\hat{R}}_{n}\\ \;\;\text{un-matricize}\;\text{the}\;\text{tensor}\;\text{X}\mathrm{(n)}\to 𝓧\\ \;\text{end}\;\text{for}\\ \;𝓒\to 𝓧 \end{array}} \end{array}$$

We then consider the complexity of TCD with random (norm sampled) subsets, which we refer to as TCD-R. The majority of complexity from each nth mode’s truncation occurs from the norm sampling of complexity $𝓞(D_{n}{\tilde{D}}_{n}^{\left(n\right)})$, and the calculation of inner products between the subset and full set ${\text{P}}_{{𝓘}_{n}}$ of complexity $𝓞({\hat{R}}_{n}D_{n}{\tilde{D}}_{n}^{\left(n\right)})$. These are then used by the significantly cheaper calculations of the coreset weights of complexity $𝓞({\hat{R}}_{n}^{3})$, and calculation of the mapping matrices ${\text{M}}_{{𝓘}_{n}}$ of complexity $𝓞({\hat{R}}_{n}^{2}D_{n})$ (re-using ${\text{P}}_{\left({𝓘}_{n},{𝓘}_{n}\right)}^{-1}$ from the coreset weights). The total complexity is thus: $𝓞(\sum_{n=1}^{N}\left(({\hat{R}}_{n}+1)D_{n}{\tilde{D}}_{n}^{\left(n\right)}+{\hat{R}}_{n}^{3}+{\hat{R}}_{n}^{2}D_{n}\right))$.

Lastly, we consider the complexity of TCD with deterministic (WKH) subsets, which we refer to as TCD-D. The majority of complexity from each nth mode’s truncation occurs from requiring calculation of ${\text{P}}_{n}={\text{X}}_{\left(n\right)}{\text{X}}_{\left(n\right)}^{⊤}\in {\mathbb{R}}^{D_{n}\times D_{n}}$, the pairwise inner products over the entire nth mode, of complexity $𝓞(D_{n}^{2}{\tilde{D}}_{n}^{\left(n\right)})$. This is followed by the WKH subset selection of complexity $𝓞({\hat{R}}_{n}^{3}D_{n})$, which yields indices ${𝓘}_{N}$ and weights ${\text{w}}_{n}$, along with the significantly cheaper calculations of the mapping matrices ${\text{M}}_{{𝓘}_{n}}$ of complexity $𝓞({\hat{R}}_{n}^{2}D_{n})$ (where we can also re-use ${\text{P}}_{\left({𝓘}_{n},{𝓘}_{n}\right)}^{-1}$ from the WKH). The total complexity is thus: $𝓞(\sum_{n=1}^{N}\left(D_{n}^{2}{\tilde{D}}_{n}^{\left(n\right)}+{\hat{R}}_{n}^{3}D_{n}+{\hat{R}}_{n}^{2}D_{n}\right))$.

Table 2 provides complexities of these methods. We note that for symmetric tensors, all methods are capable of exploiting symmetry by re-using factor matrices across symmetric modes, as described in Section IV-D. In this case, the summation $𝓞(\sum_{n=1}^{N}\left(.)\right)$ is truncated to only the number of unique modes (i.e., symmetric modes are only counted once).

In the next section, we experimentally test the TCD methods vs other efficient Tucker decomposition methods for approximating the HOSVD. We first demonstrate performance of methods on simulated data under various generative conditions. Later, we demonstrate these methods on real fMRI data in the form of functional connectivity maps (FNCs).

## V. NUMERICAL EXPERIMENTS

We first introduce the performance measures used to compare the Tensor decomposition methods. Denoting a method’s approximated tensor by $\hat{𝓧}$, the relative approximation error of $\hat{𝓧}$ is given by:

$$\text{err}(\hat{𝓧})=\frac{{\left\|𝓧-\hat{𝓧}\right\|}_{\text{F}}}{{\left\|𝓧\right\|}_{\text{F}}}\in [0\;\infty )$$

As the methods discussed in this paper are often used to approximate the HOSVD or ST-HOSVD, we also use a measure of distance between factors of the ST-HOSVD and factors of a method’s estimated HOSVD. We introduce this new measure as “HOSVD distance”, and note that its formulation utilizes the inter-symbol-interference (ISI) [111] used to evaluate the performance of blind source separation methods. Defining HOSVD distance, we denote $𝓐=\{{\text{A}}_{1},\;\ldots ,\;{\text{A}}_{N}\}$ as the N factor matrices for the “true” ST-HOSVD, and denote $\hat{𝓐}=\{{\hat{A}}_{1},\;\ldots ,\;{\hat{A}}_{N}\}$ as a method’s corresponding estimated HOSVD factor matrices, obtained by converting a method’s factorization into a HOSVD via Algorithm 3. Then the HOSVD distance between a method’s estimated HOSVD factors $\hat{𝓐}$ and the true factors 𝓐 is given by:

(16) $$\text{HOSVD}\;\text{distance}(𝓐,\hat{𝓐})=\sum_{n=1}^{N}\text{ISI(}A_{n}^{⊤}\hat{A}n)$$

where the ISI of a matrix $\text{U}\in {\mathbb{R}}^{N\times N}$ measures how close the matrix G is to a permuted diagonal matrix (a performance measure invariant to sign and permutation ambiguities of the factors), and is given by

(17) $$\text{ISI}(\text{U})=\frac{1}{2N(N-1)}[\sum_{n=1}^{N}(\sum_{m=1}^{N}\frac{\left|{\left(\text{U}\right)}_{\left(n,m\right)}\right|}{\max_{p}(\left|{\left(\text{U}\right)}_{\left(n,p\right)}\right|)}-1)\;\;\;+\sum_{m=1}^{N}(\sum_{n=1}^{N}\frac{\left|{\left(\text{U}\right)}_{\left(n,m\right)}\right|}{\max_{p}(\left|{\left(\text{U}\right)}_{\left(p,n\right)}\right|)}-1)]$$

Finally, we also measure the CPU-time of the methods. For all performance evaluations done in Sections V and VI, we use the computational resources provided by the UMBC High Performance Computing Facility (HPCF), thus CPU-time is reflective of HPCF’s capabilities.

### A. EXPERIMENTS WITH SIMULATED DATA

Our generative model of a tensor 𝓧 is as follows. For a common dimensionality across the modes D, we model a tensor $𝓧\in {\mathbb{R}}^{D\times \ldots \times D}$ as the sum of a low-rank signal tensor ${𝓧}_{S}\in {\mathbb{R}}^{D\times \ldots \times D}$ and a full-rank noise tensor ${𝓧}_{N}\in {\mathbb{R}}^{D\times \ldots \times D}$:

(18) $$𝓧={𝓧}_{S}+\eta \frac{{\left\|{𝓧}_{S}\right\|}_{\text{F}}}{{\left\|{𝓧}_{N}\right\|}_{\text{F}}}{𝓧}_{N}$$

where η is the signal to noise ratio (SNR) of 𝓧.

The signal tensor ${𝓧}_{S}$ is given in the form ${𝓧}_{S}=𝓖\times_{1}{\text{A}}_{1}\times_{2}\ldots \times_{N}{\text{A}}_{N}$, where $𝓖\in {\mathbb{R}}^{R\times \ldots \times R}$ is a core tensor for some “true core size” R, and ${\text{A}}_{n}\in {\mathbb{R}}^{D\times R}$ for n=1,…,N are the factor matrices. The core tensor 𝓖, factor matrices ${\text{A}}_{n}$, and noise tensor ${𝓧}_{N}$ are all randomly generated with entries sampled from the standard Gaussian distribution.

We consider two sets of simulated experiments: one where the generative model follows a CPD model, and one where the generative model follows a Tucker model (respectively referred to in our experiments as “CPD model data” or “Tucker model data”). These experiments use the same conditions described above except for generation of core tensor 𝓖: the Tucker experiments generate all entries of 𝓖 from the standard Gaussian distribution, whereas the CPD experiments specify 𝓖 as a superdiagonal core tensor wherein all ${\left(𝓖\right)}_{\left(i,\ldots ,i\right)}$ for i=1,…,R are drawn from the standard Gaussian distribution, and all other entries of 𝓖 equal 0.

As our paper focuses on subset-based methods for a Tucker decomposition, particularly those that approximate the HOSVD, we limit our results to variations of these methods. We thus include ST-HOSVD [89], Chidori CUR [79], [82], RST-CUR [80], a random-projection variant of HOSVD called RP-HOSVD [C] [83], [112], and another row-based tensor decomposition like those discussed in Section III-B., called RB-HOSVD [101]. While we also discuss the Fiber CUR [81] in Section III-B, we do not include Fiber CUR in our experiments as we observed poor performance compared to the other algorithms.

All of the methods and experiments are coded in MATLAB. According to the tested methods’ respective papers, we use norm sampling to obtain random row subsets of the tensor unfoldings ${\text{X}}_{\left(n\right)}$ for Chidori CUR and RB-HOSVD, and we use uniform sampling to to obtain random column subsets of ${\text{X}}_{\left(n\right)}$ for RST-CUR. Our implementation of ST-HOSVD is from the tensor toolbox version 3.6 [113], and all other methods are coded via details given in their respective papers.

To simplify the experiments, we perform all of these methods with a common “estimated core size” $\hat{R}$ that is shared across the modes of the estimated tensor. The decomposition is then converted to an estimated HOSVD decomposition that also uses the same $\hat{R}$ for all modes of the tensor. Therefore, the true HOSVD factor matrices are given by ${\text{A}}_{n}\in {\mathbb{R}}^{D\times \hat{R}}$ and the estimated factor matrices can be given by $\hat{A}n\in {\mathbb{R}}^{D\times \hat{R}}$, for n=1,…,N, in which case the U matrices in (17) are of size $\hat{R}\times \hat{R}$ . Note here that the true HOSVD is performed for some choice of estimated core size $\hat{R}$ that may differ from the true core size R of ${𝓧}_{S}$.

Under the generative model defined in (18), we vary these qualities of the model to test the methods’ performances: estimated core size $\hat{R}$, the true core sizes R, the dimensionality of the modes (mode size) D, the SNR of the simulated data tensor η, and the number of modes N. All of our experiments use these default parameters: R=4, $\hat{R}=4$, η=10, N=3, and D=200. Given the memory requirements of extremely large tensors, in the experiment that varies the number of modes, we restrict N to be either 3 or 4 modes and we use a smaller default mode size of D=80.

For all plots where we display CPU time performance, we note that these plots were essentially identical for the CPD and Tucker modeled data, thus performance was effectively independent of the generative model’s core tensor structure. Therefore, we only show the plot for the CPD model data. Additionally, we do not show figures for CPU time vs. the true core size R or the SNR η, as these experiments feature CPU times that are constant with respect to these variables.

**FIGURE 2.**
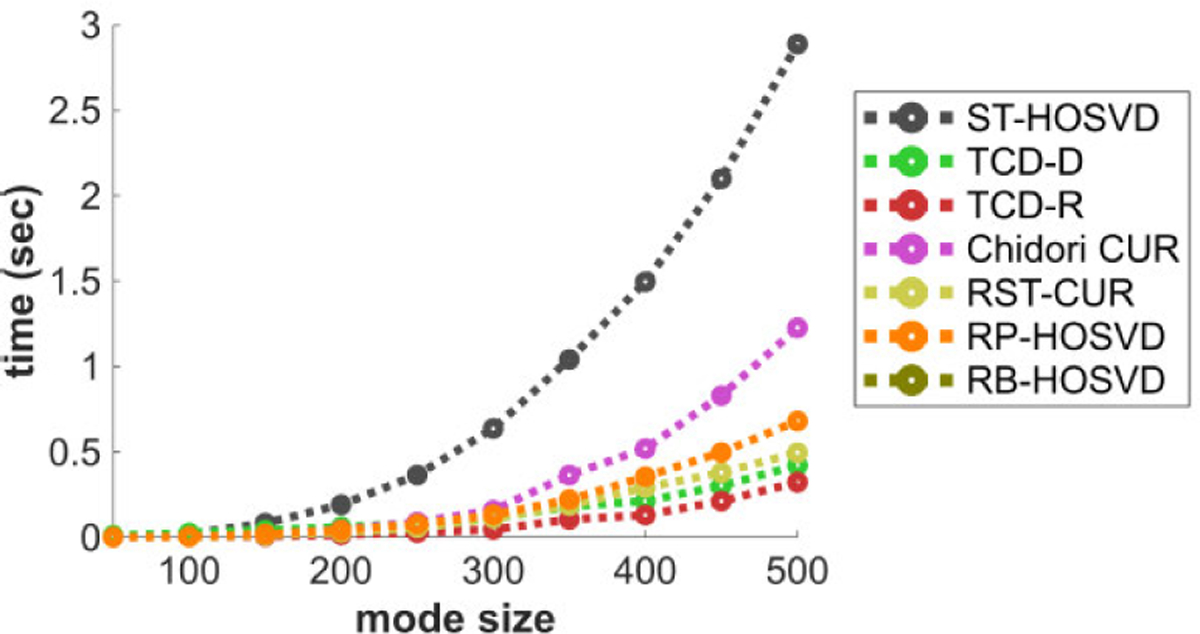
CPU time w.r.t. mode size D.

Fig. 2 plots the methods’ CPU time performance with respect to the mode size D. In this experiment ST-HOSVD is the slowest of the methods, followed by Chidori CUR, RP-HOSVD, RST-CUR, TCD-D, and TCD-R. With the default estimated core size $\hat{R}=4$, we note that TCD-D can maintain fast times in the event that $\hat{R}$ is small, which works well for tensors that have a reasonably low ranks. We also note that Chidori CUR’s slower performance is mainly due to the norm sampling over the entire tensor for each nth mode, in contrast to sampling over truncated tensors such as done in other methods. Chidori CUR is significantly faster when uniform sampling is done in place of norm sampling, with an accompanying degree of loss in approximation performance.

**FIGURE 3.**
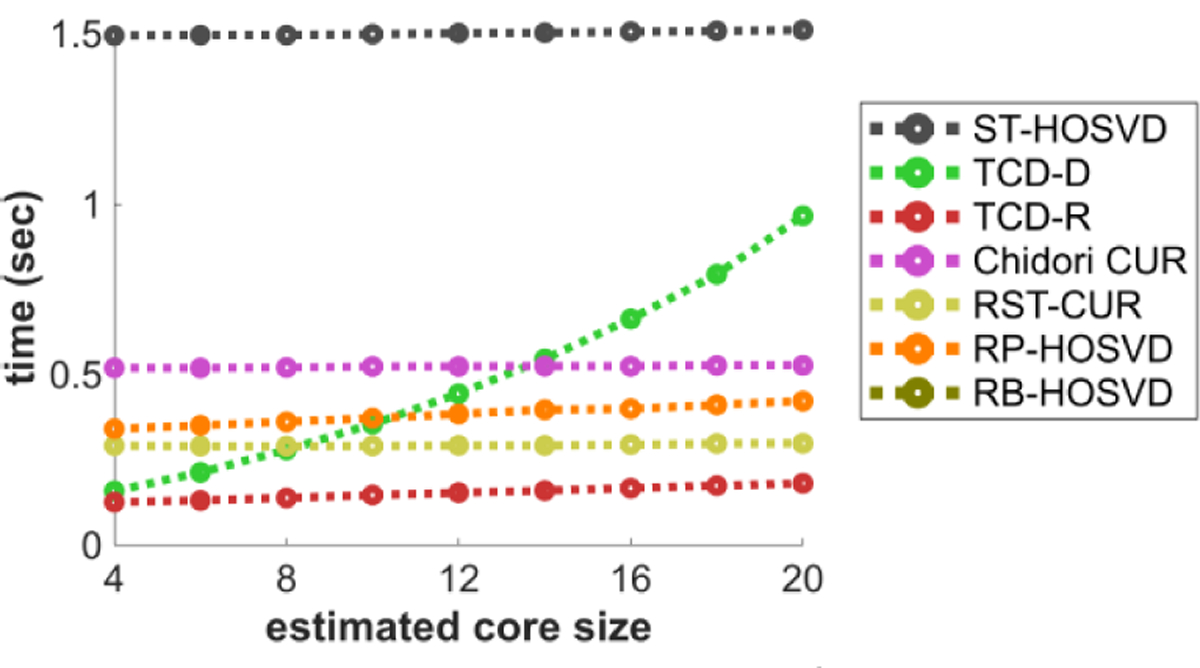
CPU time w.r.t. estimated core size $\hat{R}$.

Fig. 3 plots the methods’ CPU time performance with respect to the estimated core size $\hat{R}$. TCD-D faces larger complexity with higher R, whereas all other methods have complexity that only increases slightly with increasing R. This may motivate other methods besides TCD-D for when CPU time is a priority and larger $\hat{R}$ are desired. However, TCD-D is still unique among these methods for deterministically selecting elements from the modes. Thus, compared to these otherwise predominately randomized methods, TCD-D is perhaps unique in its utility for feature selection.

**FIGURE 4.**
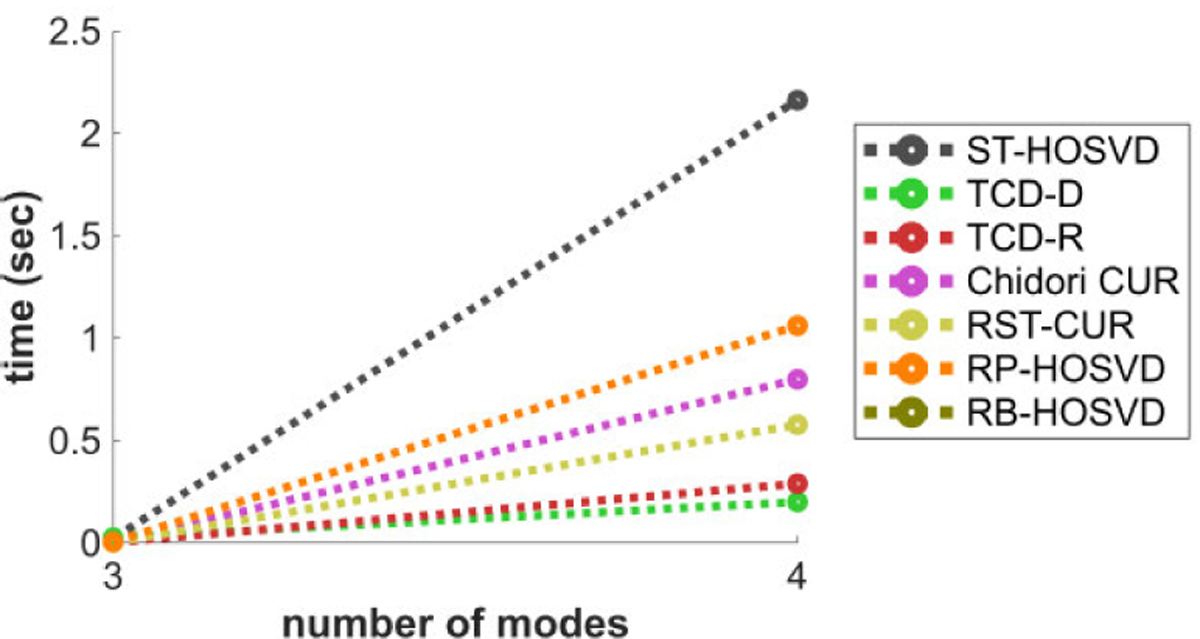
CPU time w.r.t. number of modes N.

Fig. 4 plots the methods’ CPU time performance with respect to the number of modes N, for N=3 and N=4. TCD-D and TCD-R are among the fastest methods in this experiment, and interestingly, TCD-D is the fastest despite being deterministic. We observe that this is due to how MATLAB’s efficiency varies with respect to different mathematical operations: MATLAB is especially efficient in computing the Gram matrix ${\text{X}}_{\left(n\right)}{\text{X}}_{\left(n\right)}^{⊤}\in {\mathbb{R}}^{D\times D}$, so much so that it can actually be more efficient to compute ${\text{X}}_{\left(n\right)}{\text{X}}_{\left(n\right)}^{⊤}$ than even the fastest methods for calculating norms of rows of ${\text{X}}_{\left(n\right)}$, which is required of the norm sampling approaches like TCD-R, Chidori CUR, and RB-HOSVD. Depending on the efficiency of the calculations, the programming environment used and the dimensions of the tensor, these methods may benefit by using ${\text{X}}_{\left(n\right)}{\text{X}}_{\left(n\right)}^{Τ}$ to calculate the norms. At the same time, this also demonstrates the efficiency of the WKH procedure in TCD-D for smaller $\hat{R}$, since it does not lead to significant increases in complexity above the other methods.

**FIGURE 5.**
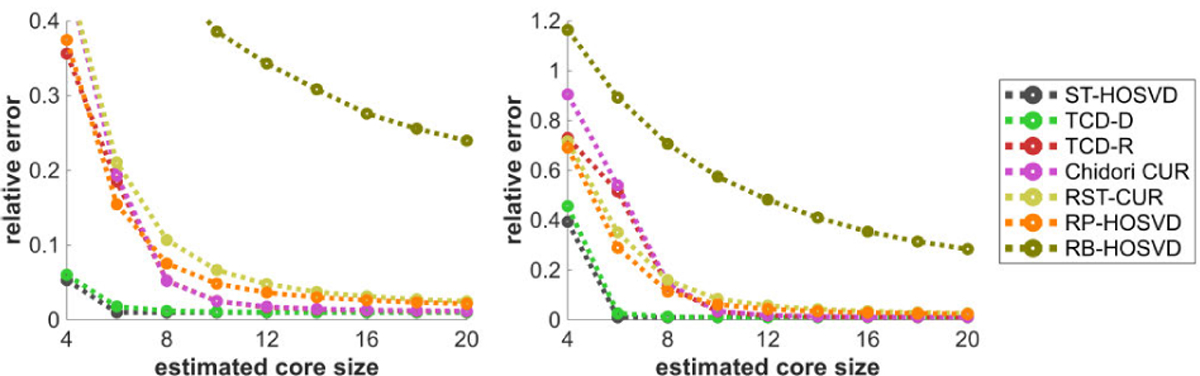
Relative error w.r.t. the estimated core size $\hat{R}$. Left: CPD model data. Right: Tucker model data.

Fig. 5 plots the methods’ relative error performance with respect to the estimated core size $\hat{R}$. All methods’ decompositions exponentially approach the true tensor in approximation quality with diminishing returns in $\hat{R}$ . Performance of TCD-R in this experiment is comparable to Chidori CUR, with these methods only beaten by ST-HOSVD and TCD-D for lower $\hat{R}$.

**FIGURE 6.**
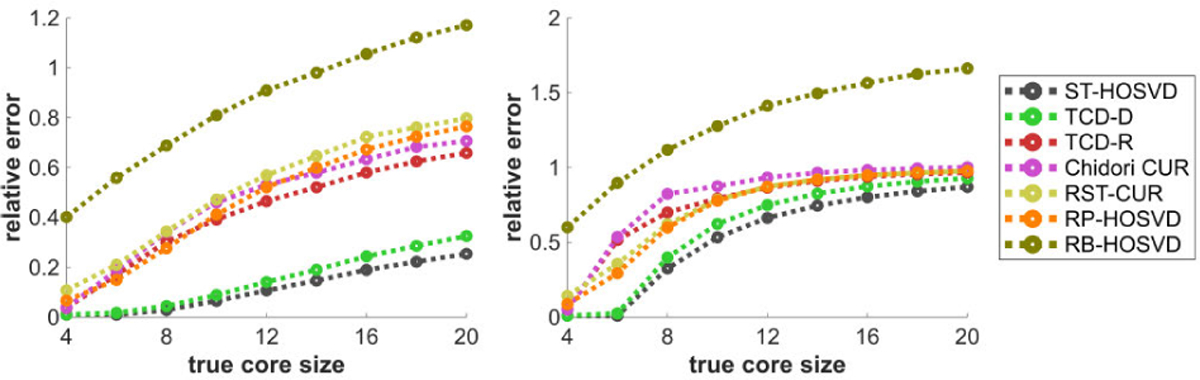
Relative error w.r.t. the true core size R. Left: CPD model data. Right: Tucker model data.

Fig. 6 plots the methods’ relative error performance with respect to the true core size R. Given a fixed estimated core size $\hat{R}=4$, all decompositions perform worse as the true core size $\hat{R}$ increases, where with R>4 the decompositions are effectively underparametrizing and/or undersampling their model of the tensor. Like in Fig. 5, in this experiment TCD-D has an estimation performance that is only slightly worse than ST-HOSVD. After these methods, TCD-R has the third best performance, exceeding that of Chidori CUR for larger $\hat{R}$.

**FIGURE 7.**
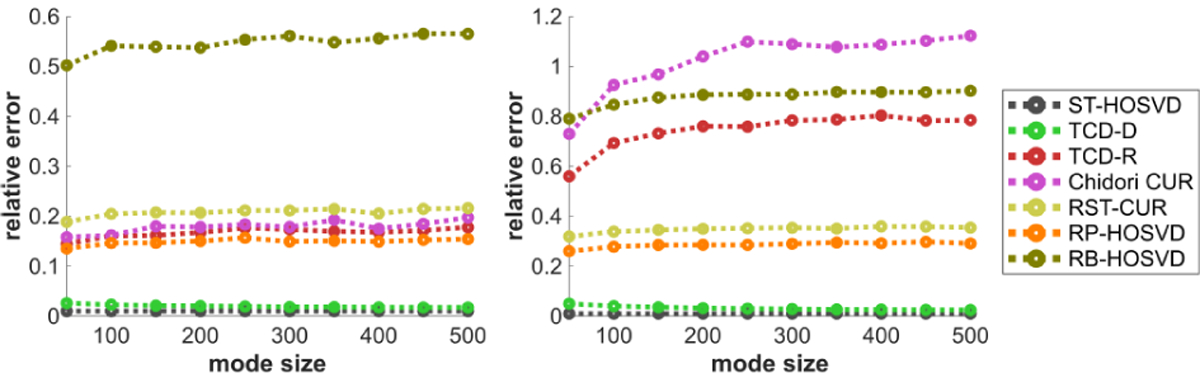
Relative error w.r.t. the mode size D. Left: CPD model data. Right: Tucker model data.

Fig. 7 plots the methods’ relative error performance with respect to the mode size D. These performances are mostly constant in D with the CPD data (left), but for the Tucker data, some methods like Chidori CUR and TCD-R feature slightly worse performances with larger D, up to diminishing returns. Most of the randomized methods have much more comparable relative errors for the CPD model data, with significantly higher spread with the Tucker model data.

**FIGURE 8.**
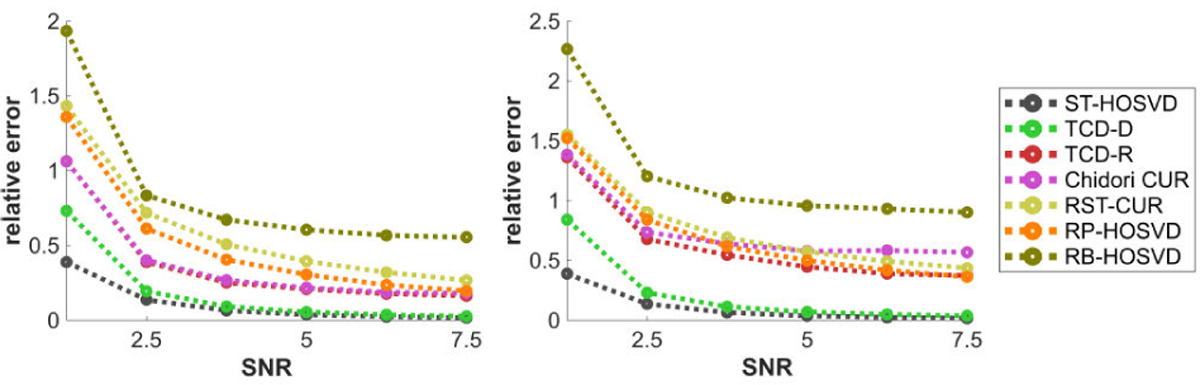
Relative error w.r.t. the SNR η. Left: CPD model data. Right: Tucker model data.

Fig. 8 plots the methods’ relative error performance with respect to the signal to noise ratio (SNR) η. Subject to diminishing returns, all methods perform significantly better in approximating the tensor with higher SNR, and TCD-D and TCD-R appear to provide some of the better approximations with lower SNR values. With higher SNR values, TCD-D’s performance is comparable to ST-HOSVD and TCD-R’s performance is comparable to Chidori CUR.

**FIGURE 9.**
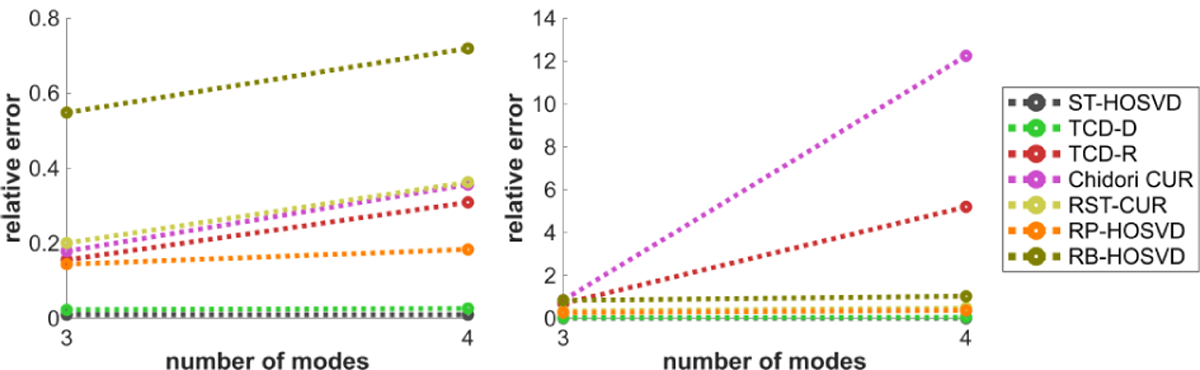
Relative error w.r.t. the number of modes N. Left: CPD model data. Right: Tucker model data.

Fig. 9 plots the methods’ relative error performance with respect to the number of modes N, for N=3 and N=4. An apparent disadvantage to Chidori CUR and TCD-R occurs when N=4, in which case these methods’ performances appear to suffer considerably, whereas all other methods are not as much affected by change of N.

We now discuss the methods’ performances in terms of the HOSVD distance measure defined in (17). We note that we compare each algorithm’s estimated HOSVD factors to the “true” factors estimated by ST-HOSVD for the same choice of $\hat{R}$, thus we don’t include ST-HOSVD in these plots since it has a HOSVD distance of 0 with itself.

**FIGURE 10.**
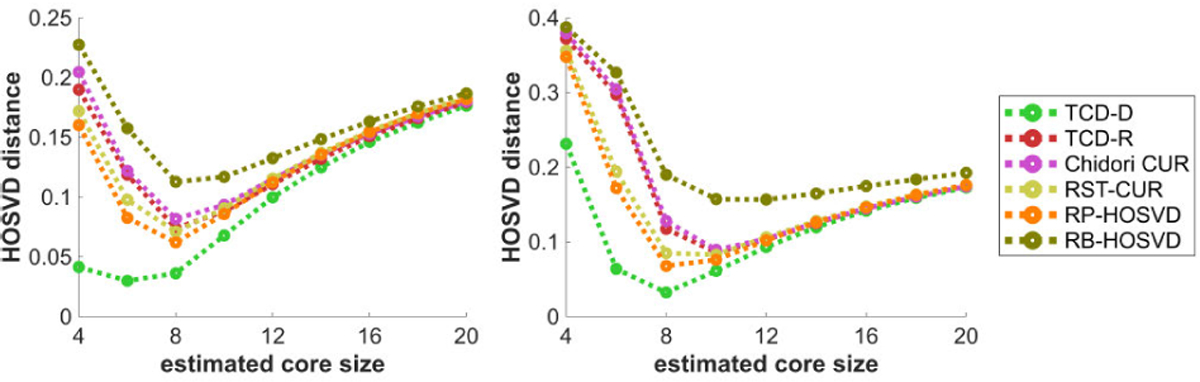
HOSVD distance w.r.t. the estimated core size $\hat{R}$. Left: CPD model data. Right: Tucker model data.

Fig. 10 plots the methods’ HOSVD distances with respect to the estimated core size $\hat{R}$. All plots feature a clear U-shaped performance curve where the best performance generally occurs at $\hat{R}=6$, slightly higher than the true core size R=4. Interestingly, these U-shaped HOSVD distance vs. $\hat{R}$ plots are notably different in shape from the monotonically decreasing error vs. $\hat{R}$ plots in Fig. 5. While the relative error of the decompositions only decreases when the decompositions model allows for more complexity (via increasing $\hat{R}$ ), the HOSVD distance represents more of a measure of *parameter estimation*, where the desired parameters are the true ST-HOSVD factors, and are best estimated when the estimated number of factors $\hat{R}$ is close to the true number R.

**FIGURE 11.**
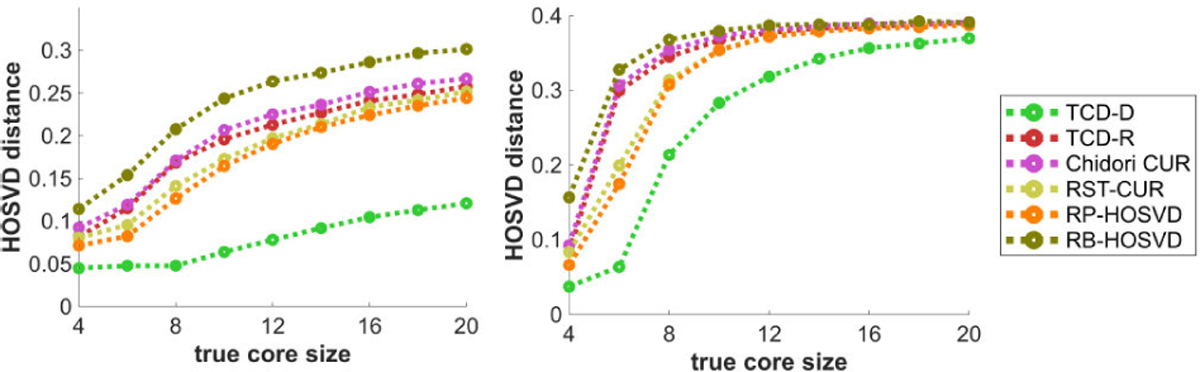
HOSVD distance w.r.t. the true core size R. Left: CPD model data. Right: Tucker model data.

Fig. 11 plots the methods’ HOSVD distances with respect to the true core size R. Whereas Fig. 10 shows a U-shaped curve with varying $\hat{R}$, Fig. 11 shows that increasing R strictly worsens the methods’ performances as $R<\hat{R}$ for a fixed $\hat{R}=4$. All methods perform poorly when R is too large for the Tucker model data. However with the CPD model data, TCD-D performs significantly better than all other methods, especially with large R.

**FIGURE 12.**
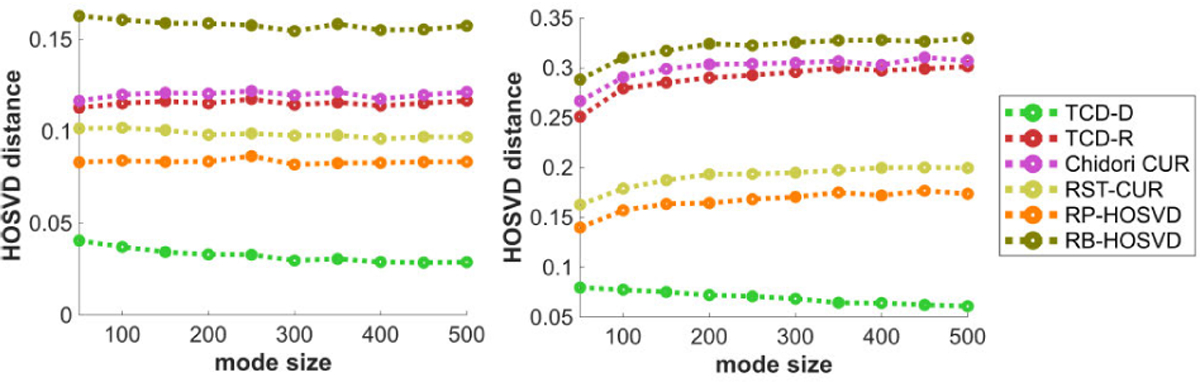
HOSVD distance w.r.t. the mode size D. Left: CPD model data. Right: Tucker model data.

Fig. 12 plots the methods’ HOSVD distances with respect to the mode size D. Like in Fig. 7, performances are mostly constant in D with the CPD data (left), but for the Tucker data, all methods except TCD-D feature slightly worse performances with larger D, whereas TCD-D actually features slightly better performances for larger D, up to diminishing returns. We suspect the reason for TCD-D actually doing better for larger D is that as all other variables are fixed, the tensor is generated the same with different D but there are just more elements available to consider subsets over, in which case TCD-D’s deterministic WKH has more options of a subset that better minimize the discrepancy measure, and thus better match the PCs of the tensor.

**FIGURE 13.**
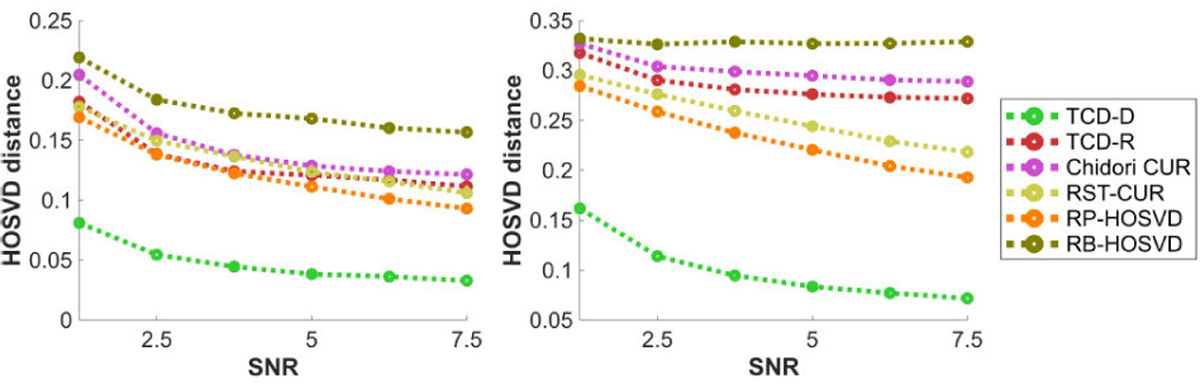
HOSVD distance w.r.t. the SNR η. Left: CPD model data. Right: Tucker model data.

Fig. 13 plots the methods’ HOSVD distances with respect to the SNR η. Like in Fig. 8, subject to diminishing returns, all methods perform significantly better with higher SNR, with TCD-R’s performance slightly better than Chidori CUR but typically worse than RST-CUR and RP-HOSVD. Whereas in Fig. 8 all methods’ relative errors nearly converge to 0 with increased SNR, TCD-D’ HOSVD distance in Fig. 13 converges significantly faster to 0 with increased SNR than the other methods’ HOSVD distances.

**FIGURE 14.**
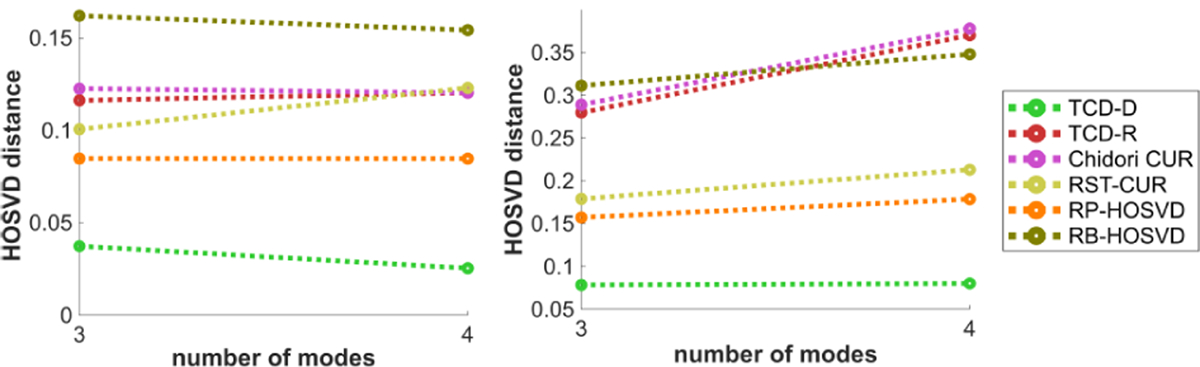
HOSVD distance w.r.t. the number of modes N. Left: CPD model data. Right: Tucker model data.

Fig. 14 plots the methods’ HOSVD distances with respect to the number of modes N, for N=3 and N=4. Like in Fig. 9, TCD-R Chidori CUR perform worse with N=4 with the Tucker model data, whereas all other methods’ HOSVD distances are not as much affected by N.

To summarize these experiments, we observe that TCD-R is among the most efficient of these methods, and TCD-D is also efficient when $\hat{R}$ is small. In most experiments, TCD-R yields comparatively better approximation error and HOSVD distance performance vs. other methods with similar time complexities. Furthermore, TCD-D’s performance is typically significantly better than all other tested methods, and even competes closely to that of ST-HOSVD despite using only a subset of the tensor’s elements.

In the next section, we perform these methods on real data in the form of fMRI functional connectivity matrices (FNCs), where we visually demonstrate performance of these methods and also demonstrate the use of TCD-D for feature selection.

### B. EXPERIMENT WITH FMRI DATA

Our experiments use resting-state fMRI data from the bipolar-schizophrenia network on intermediate phenotypes (B-SNIP) [114], [115], where our data tensor 𝓧 was obtained from the acquisition and preprocessing steps described in [116] and [117]. The main goals of these experiments are to:

- Demonstrate performance of the tensor decomposition methods on real fMRI data in terms of estimation quality and computational efficiency.
- Demonstrate TCD-D’s ability (unique among these methods) to perform feature selection within modes, selecting well-representative elements of the data. In our case, these elements are functional networks (FNs) which are typically used to characterize neurological phenomenon.

We now detail how the data tensor 𝓧 was formed. The fMRI dataset includes 176 healthy control and 176 schizophrenia patients for a total of K=352 subjects. The data was first preprocessed and then analyzed via constrained independent vector analysis (cIVA) to extract meaningful latent factors for describing the data. From each subject’s data, 53 spatial factors were extracted which correspond to biologically important functional networks (FNs). These factors are representative of seven different functional domains: subcortical (SC, 5 FNs), auditory (AUD, 2 FNs), sensorimotor (MOT, 9 FNs), visual (VIS, 9 FNs), cognitive control (CC, 17 FNs), default mode (DMN, 7 FNs) and cerebellar (CB, 4 FNs). Corresponding to each of these 53 spatial factors are time course factors, representing amplitudes of the networks at each point of measurement, and the correlations between these time courses are particularly useful for representing relationships between the networks. All pairwise Pearson correlations between any two of the 53 networks’ time courses is represented in a symmetric 53 × 53 matrix called a functional network connectivity (FNC) matrix. Our experiment constructs these FNC matrices across each of 352 subjects, and forms an FNC tensor $𝓧\in {\mathbb{R}}^{53\times 53\times 352}$.

A key factor in dealing with the data tensor is understanding its effective *n*-ranks given how the tensor was obtained. Our FNC data was extracted from functional networks that are expected to be maximally statistically independent from one another, being extracted from cIVA which maximizes statistical independence between networks. Therefore, we expect low correlation between the spatial components of different networks, and this can also result in time courses that demonstrate low correlation between disparate networks. This results in a tensor with effectively high *n*-ranks, thus decompositions of FNC tensors like 𝓧 require higher numbers of factors ${\hat{R}}_{n}$ to adequately approximate the FNCs. This presents a challenge for the decomposition methods to approximate the tensor with relatively fewer factors, allowing us to better magnify and compare the methods’ estimation capabilities.

Due to the higher *n*-ranks of the FNC tensor, we test the algorithms on two different forms of the FNC tensor: one being the original FNC tensor, and the other being the elementwise squaring of the FNC tensor. The elementwise squaring provides R-squared values representing the degree of association between the network time courses. Taking the elementwise square of these FNCs effectively increases the spread of the singular values of each mode unfolding ${\text{X}}_{\left(n\right)}$, allowing for better approximation with lower-rank models while still maintaining an interpretable decomposition.

For our experiments, we did a prior exploratory analysis over several candidates of estimated numbers of factors ${\hat{R}}_{n}$, and ultimately implemented $\left[{\hat{R}}_{1},{\hat{R}}_{2},{\hat{R}}_{3}]=[20,20,352\right]$ for both forms of the tensor. The reasoning for these choice of ${\hat{R}}_{n}$ were as follows: to better exemplify the approximation quality differences between the methods, to reasonably approximate the FNCs without too many factors, and to provide a more parsimonious model which TCD-D can then use to select networks whose R-squared values are “well representative” of all R-squared values in 𝓧. These 20 networks could then be interpreted as particularly informative for approximating the relationships between any of the 53 networks.

We use the same tensor decomposition methods in Section V-A to decompose our FNC tensor 𝓧. In order to also exploit the symmetry of 𝓧, we modify each of these methods to use the same symmetry exploiting process described at the end of Section IV-D. Therefore, since the first and second modes are symmetric (pertaining to the 53 networks), the same factor matrix is used for both of these modes, and the core tensor is thus also symmetric with respect to these modes.

As done in the previous section, our experiments measure performance via CPU time, relative error, and HOSVD distance. Additionally, we implement a measure of how consistent the methods’ approximated HOSVD decompositions are with respect to different runs of the decompositions, which corresponds to different random subsets per run for the randomized methods. In defining this measure, we denote ${\hat{𝓐}}^{\left[m\right]}$ as the approximated HOSVD factors from a mth run of a decomposition method over the data, and define the set of the ${\hat{𝓐}}^{\left[m\right]}$ across M runs by the set $𝓕=\{{\hat{𝓐}}^{\left[1\right]},\;\ldots ,\;{\hat{𝓐}}^{\left[M\right]}\}$. Then our measure of “cross-distance”, the average distance between any two runs of a decomposition, is given by:

(19) $$\text{cross-distance(}𝓕\text{)=}\frac{\sum_{\begin{array}{l} m_{1}=1\\ m_{2}=1 \end{array}}^{M}\text{HOSVD}\;\text{distance}\;({\hat{𝓐}}^{\left[m_{1}\right]},{\hat{𝓐}}^{\left[m_{2}\right]})}{M^{2}}.$$

This “cross-distance” can be considered a generalization of the “cross-ISI” measure used to measure distances between runs for Blind Source Separation (BSS) methods [118].

Along with using cross-distance to measure the variability of the randomized methods, we also use cross-distance to obtain a single run that is the most well representative of all other runs, for which we may plot the FNCs approximated by this run to visually compare the average approximation quality of the methods. The plotted average FNCs were obtained by constructing the most representative run’s approximate tensor $\hat{𝓧}$ from its factorization, and then averaging the approximated subject FNCs across the 352 subjects.

**FIGURE 15.**
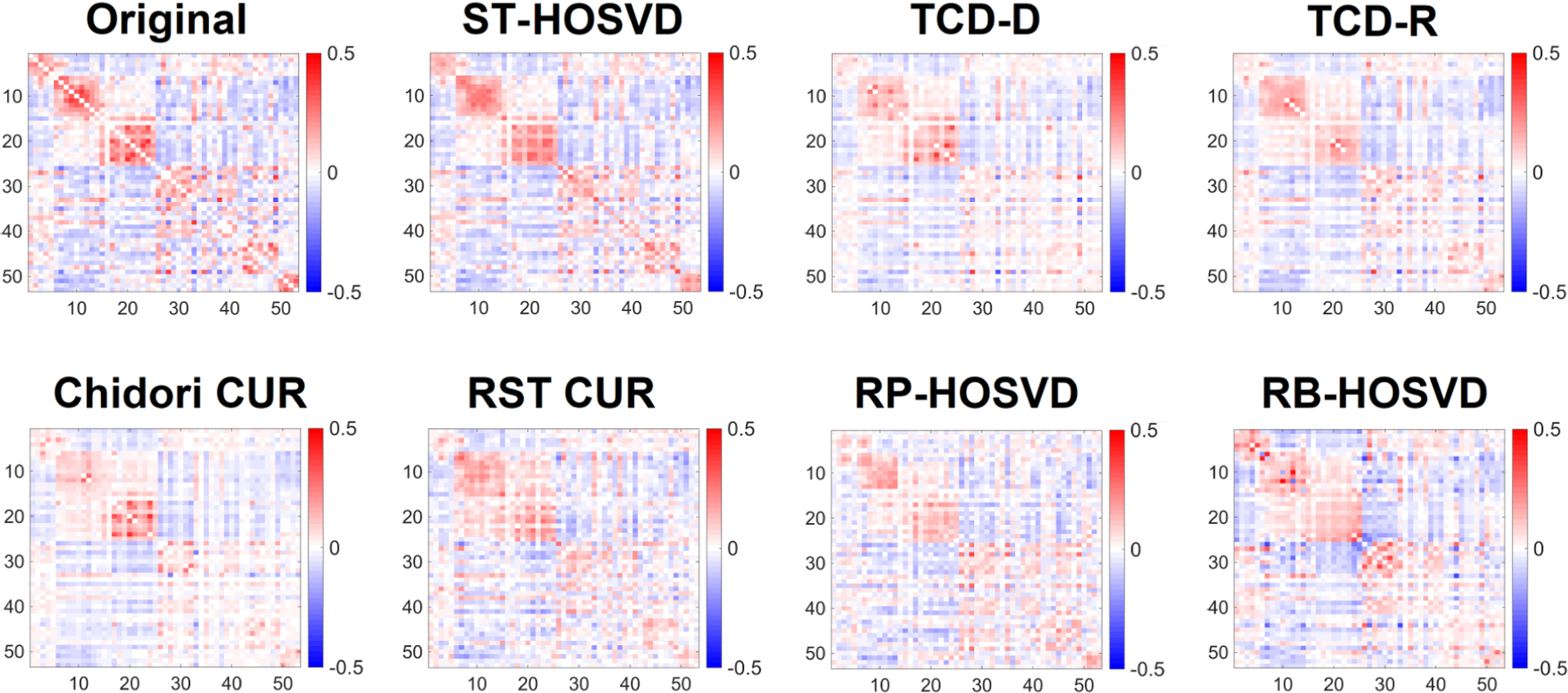
Plots of the average FNCs obtained by the approximated original FNC tensor $\hat{𝓧}$, for each method’s most typical run (the run with the minimum cross-distance to all other runs). All methods used the ranks $\left[{\hat{R}}_{1},{\hat{R}}_{2},{\hat{R}}_{3}]=[20,20,352\right]$.

**FIGURE 16.**
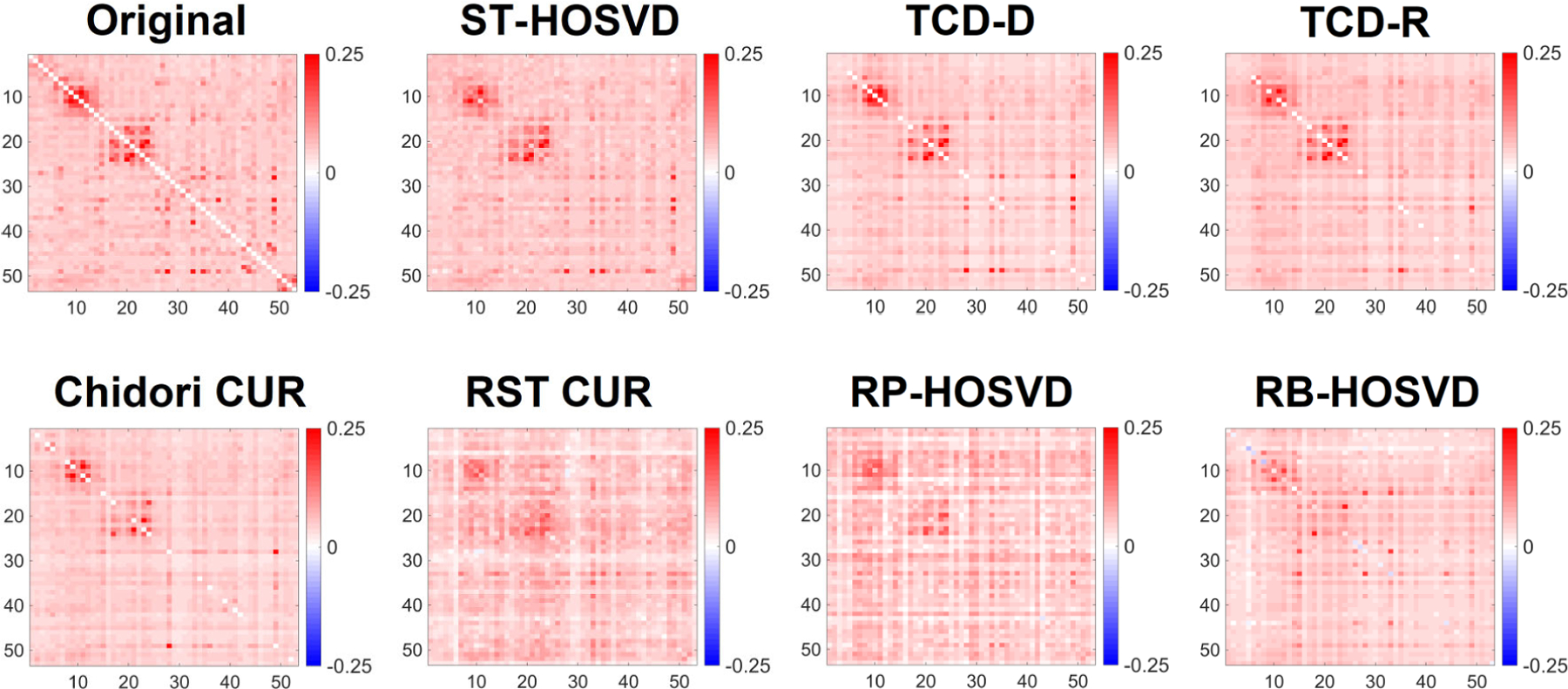
Plots of the average FNCs obtained by the approximated elementwise squared FNC tensor $\hat{𝓧}$, for each method’s most typical run (the run with the minimum cross-distance to all other runs). All methods used the ranks $\left[{\hat{R}}_{1},{\hat{R}}_{2},{\hat{R}}_{3}]=[20,20,352\right]$.

Fig. 15 and Fig. 16 exhibit the average FNCs extracted from a typical run of each method, on the FNC tensor and squared FNC tensor respectively. In both forms of the data, the FNCs typically feature two well-defined blocks on the diagonal. These correspond to the motor (upper block) and visual (lower block) groups of networks, which feature high correlation and R-squared values within the groups. Because of the larger degree of association within these networks, their larger values in 𝓧 lead them to be especially important for approximating 𝓧. Viewing the averages of FNCs in Fig. 15, we observe all methods are able to reasonably approximate at least one of these blocks, with ST-HOSVD, TCD-D, TCD-R, Chidori CUR, and RP-HOSVD demonstrating the two well-defined blocks, and TCD-D and TCD-R having performance closest to ST-HOSVD. Viewing the average of squared FNCs in Fig. 16, we note that all methods except for RB-HOSVD demonstrate two clearly defined blocks, with TCD-D and TCD-R having performance closest to ST-HOSVD.

Tables 3 and 4 presents each method’s performance measures on the FNC tensor. All methods provide relatively higher relative errors, as the decomposition ranks $\left[{\hat{R}}_{1},{\hat{R}}_{2},{\hat{R}}_{3}]=[20,20,352\right]$ are perhaps relatively conservative for the more heterogeneous nature of the FNC tensor. While in practice we select ${\hat{R}}_{n}$ to provide an approximation quality that is nearly identical to the original tensor, our choice of lower ${\hat{R}}_{n}$ is useful for better magnifying the approximation capabilities of the methods, which are clearly demonstrated in the much wider range of their values. ST-HOSVD provides the best relative error, and TCD-D features a comparatively similar error while simultaneously identifying representative networks. Among the more efficient methods, RST-CUR is the fastest method but has the second worst error and worst cross-distance, whereas TCD-R is the second fastest method with the third lowest error, HOSVD distance, and cross-distance. This demonstrates that TCD-D and TCD-R provide good performance measures given their time complexities, and can provide reasonably good approximations to the tensor with fewer factors ${\hat{R}}_{n}$.

Additionally, a key distinction between TCD-D and the other methods is that TCD-D deterministically selects elements that are well representative of the tensor. Thus, TCD-D is unique among these methods for the capability of performing feature selection with the tensor data. With this fMRI dataset, TCD-D deterministically selects a reasonably “best” subset of the factor networks. We now consider the interpretation of the TCD-D selected networks. We observed that several TCD-D selected networks were selected not only for the 20 selected networks of the original FNC data, but also the 20 selected networks of the elementwise squared data, highlighting the importance of these networks (a total of 14 networks shared between the two forms of the tensor, corresponding to the indices 5, 8, 9, 12, 15, 17, 23, 24, 27, 28, 33, 45, 49, 51). Table 5 overviews details of these 14 networks identified over both forms of the data tensor, including their associated factor index in the FNCs (their index $i_{1}=i_{2}$ in 𝓧), the region of the brain the network corresponds to, and the group of networks it associates with.

These identified networks, including regions such as the thalamus, superior temporal gyrus, superior frontal gyrus, and posterior cingulate cortex, are significant as they represent crucial functional “blocks of networks” within the brain. Each of these networks is associated with specific functional domains, such as sensorimotor (e.g., left postcentral gyrus, superior parietal lobule), visual (e.g., inferior occipital gyrus), cognitive control (e.g., inferior parietal lobule), and the default mode network (e.g., posterior cingulate cortex). Clinically, these functional networks have been reported as significant brain regions highly associated with various psychiatric disorders. For instance, the superior frontal gyrus and posterior cingulate cortex have been identified in previous research as valuable biomarkers for different psychiatric conditions [116], [119], [120], [121], [122]. Furthermore, the fact that 14 of the 20 networks were identified over both forms of the tensor (original FNCs, and elementwise squared FNCs) demonstrates robustness of the proposed TCD-D method, showing consistent identification of meaningful functional areas that are associated with several psychiatric disorders. For example, reduced connectivity between the posterior cingulate and frontal areas in patients with first-episode schizophrenia has been reported in [123]. The failure of appropriate posterior cingulate cortex deactivation has been reported as potential biomarker in traumatic brain injury and mental disorders like ADHD, autism and schizophrenia [124].

## VI. CONCLUSION

This paper presents efficient Tucker decomposition methods via using a small subtensor as a multilinear basis over the full data tensor, which we refer to as tensor coreset decompositons (TCD). The methods operate by sequentially truncating the tensor by replacing it with a *coreset* of elements from one or more of the tensor’s modes, with the coreset calculated such that it minimizes a discrepancy between itself and the HOSVD core tensor: principal components of the tensor’s unfoldings. This process sub-sequentially estimates mapping matrices that serve as the decomposition’s factor matrices, which can also be useful for efficiently approximating the tensor’s HOSVD.

For quantifying the “representativeness” of a coreset over the data tensor, we introduced a discrepancy-based measure that has straightforward connections to the cost function of HOSVD. We use this measure to develop a new efficient nonnegative least squares (NNLS) procedure for selecting the coreset weights, such that we minimize the discrepancy with respect to a choice of subset.

For decompositions that put greater emphasis on efficiency, we proposed “TCD-R” which randomly selects the subsets using norm sampling. For decompositions that place greater emphasis on approximation quality, and utility of selecting well representative subsets and for feature selection, we proposed “TCD-D” which uses a deterministic subset selection scheme based on the method of weighted kernel herding (WKH). Compared to previous methods, TCD-D is notably unique for its ability to perform unsupervised feature selection within the modes of the tensor data.

Finally, we experimentally demonstrate that our methods generally provide good balances between efficiency, approximation error quality, and quality of factors when converted to a HOSVD. Furthermore, we demonstrate on real fMRI FNC data that TCD-D is able to identify meaningful subsets of functional networks which are able to well-approximate the relationships between all networks in the FNC tensor.

## Figures and Tables

**TABLE 1.** Notation used in this paper.

Notation	Definition
x	scalar
x	vector
X	matrix
𝓧	tensor
N	number of modes
$D_{n}$	dimensionality of nth mode, for n=1,…,N
$R_{n}$	*n*-rank: rank of ${\text{X}}_{\left(n\right)}$
${\hat{R}}_{n}$	number of factors in decomposition of nth mode
${\tilde{D}}_{n}$	$\prod_{m=1}^{N}D_{m}$
${\tilde{D}}_{n}^{\left(n\right)}$	$\left(\prod_{m=1}^{n-1}{\hat{R}}_{m})\;(\prod_{m=n+1}^{N}D_{m}\right)$
${\hat{T}}_{n}$	$\prod_{\begin{array}{l} m=1\\ m\neq n \end{array}}^{N}{\hat{R}}_{m}$
$i_{n}$	ith index of the *n*th mode, for $i_{n}=1,\ldots ,D_{n}$
${𝓘}_{n}$	index set of *n*th mode (with cardinality ${\hat{R}}_{n}$)
$𝓘=\{{𝓘}_{1},\;\ldots ,\;{𝓘}_{N}\}$	All modes’ index sets
${\left(𝓧\right)}_{\left(:,\ldots ,i_{n},\ldots ,:\right)}$	element $i_{n}$ of the nth mode
${\left(𝓧\right)}_{\left(:,\ldots ,{𝓘}_{n},\ldots ,:\right)}$	subset of nth mode corresponding to indices ${𝓘}_{n}$
${\text{X}}_{\left(n\right)}$	nth mode unfolding of 𝓧
$𝓧\times_{n}{\text{A}}_{n}$	nth mode tensor product of 𝓧 with ${\text{A}}_{n}$
${\text{X}}^{⊤}$	matrix transpose
${\text{X}}^{†}$	matrix pseudoinverse
$\text{X}{\text{X}}^{†}$	projection matrix
${\text{M}}_{{𝓘}_{n}}$	nth mode “mapping” matrix corresponding to ${𝓘}_{n}$

**TABLE 2.** Computational complexities of TCD and similar methods described in Sections II and III. For truncated methods, we assume that the truncation order is n=1,…,N.

ST-HOSVD	$𝓞(\sum_{n=1}^{N}\min(D_{n}^{2}{\tilde{D}}_{n}^{\left(n\right)},{\left({\tilde{D}}_{n}^{\left(n\right)}\right)}^{2}D_{n}))$
Chidori CUR	$𝓞(\sum_{n=1}^{N}\left(D_{n}{\tilde{D}}_{n}+{\hat{R}}_{n}D_{n}{\hat{T}}_{n}+{\hat{R}}_{n}^{3}+{\hat{R}}_{n}^{2}D_{n}\right))$
TCD-R	$𝓞(\sum_{n=1}^{N}\left(({\hat{R}}_{n}+1)D_{n}{\tilde{D}}_{n}^{\left(n\right)}+{\hat{R}}_{n}^{3}+{\hat{R}}_{n}^{2}D_{n}\right))$
TCD-D	$𝓞(\sum_{n=1}^{N}\left(D_{n}^{2}{\tilde{D}}_{n}^{\left(n\right)}+{\hat{R}}_{n}^{3}D_{n}+{\hat{R}}_{n}^{2}D_{n}\right))$

**TABLE 3.** Performances of methods on the original FNC tensor $𝓧\in {\mathbb{R}}^{53\times 53\times 352}$, averaged over 1000 independent runs over the data. Best performances per measure are bolded.

	CPU-time (sec)	relative error	HOSVD distance	cross-distance
ST-HOSVD	0.026	**0.500**	**0**	**0**
TCD-D	0.025	0.650	0.128	**0**
TCD-R	0.005	0.686	0.130	0.145
Chidori CUR	0.007	0.687	0.133	0.150
RST-CUR	**0.002**	0.691	0.132	0.169
RP-HOSVD	0.006	0.689	0.207	0.169
RB-HOSVD	0.015	0.940	0.148	0.163

**TABLE 4.** Performances of methods on the elementwise squared FNC tensor $𝓧\in {\mathbb{R}}^{53\times 53\times 352}$, averaged over 1000 independent runs over the data. Best performances per measure are bolded.

	CPU-time (sec)	relative error	HOSVD distance	cross-distance
ST-HOSVD	0.026	**0.384**	**0**	**0**
TCD-D	0.025	0.464	0.150	**0**
TCD-R	0.005	0.514	0.155	0.155
Chidori CUR	0.007	0.520	0.160	0.157
RST-CUR	**0.002**	0.549	0.156	0.185
RP-HOSVD	0.006	0.528	0.218	0.175
RB-HOSVD	0.015	0.890	0.176	0.183

**TABLE 5.** Descriptions of 14 factors selected by TCD-D, shared between the 20 selected from the original FNC tensor and the 20 from the elementwise-squared FNC tensor.

Index	Region	Network	Component
5	Thalamus	subcortical (SC)	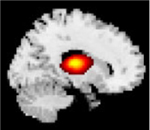
8	Postcentral gyrus	sensorimotor (SM)	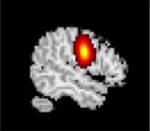
9	Left postcentral gyrus	sensorimotor (SM)	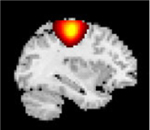
12	Superior parietal lobule	sensorimotor (SM)	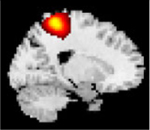
15	Superior parietal lobule	sensorimotor (SM) (SM)	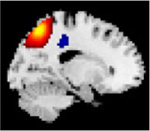
17	Calcarine gyrus	visual (VIS)	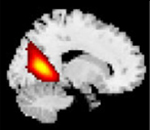
23	Inferior occipital	gyms visual (VIS)	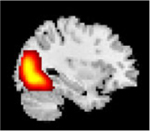
24	Lingual gyms	visual (VIS)	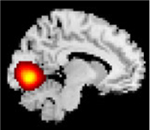
27	Inferior parietal lobule	sensorimotor (SM)	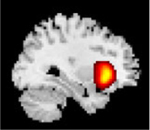
28	Superior frontal gyms	cognitive control (CC)	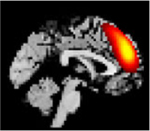
33	Inferior parietal lobule	sensorimotor (SM)	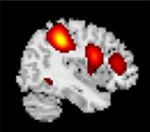
45	Anterior cingulate cortex	default-mode network (DMN))	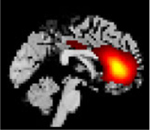
49	Posterior cingulate cortex	default-mode network (DMN)	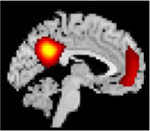
51	Cerebellar	Cerebellar (CB)